# Base Composition, Codon Usage, and Patterns of Gene Sequence Evolution in Butterflies

**DOI:** 10.1093/gbe/evad150

**Published:** 2023-08-11

**Authors:** Karin Näsvall, Jesper Boman, Venkat Talla, Niclas Backström

**Affiliations:** Evolutionary Biology Program, Department of Ecology and Genetics (IEG), Uppsala University, Uppsala, Sweden; Evolutionary Biology Program, Department of Ecology and Genetics (IEG), Uppsala University, Uppsala, Sweden; Evolutionary Biology Program, Department of Ecology and Genetics (IEG), Uppsala University, Uppsala, Sweden; Evolutionary Biology Program, Department of Ecology and Genetics (IEG), Uppsala University, Uppsala, Sweden

**Keywords:** comparative genomics, codon usage bias, GC-biased gene conversion, substitution rates, tRNA genes, *Leptidea*

## Abstract

Coding sequence evolution is influenced by both natural selection and neutral evolutionary forces. In many species, the effects of mutation bias, codon usage, and GC-biased gene conversion (gBGC) on gene sequence evolution have not been detailed. Quantification of how these forces shape substitution patterns is therefore necessary to understand the strength and direction of natural selection. Here, we used comparative genomics to investigate the association between base composition and codon usage bias on gene sequence evolution in butterflies and moths (Lepidoptera), including an in-depth analysis of underlying patterns and processes in one species, *Leptidea sinapis*. The data revealed significant G/C to A/T substitution bias at third codon position with some variation in the strength among different butterfly lineages. However, the substitution bias was lower than expected from previously estimated mutation rate ratios, partly due to the influence of gBGC. We found that A/T-ending codons were overrepresented in most species, but there was a positive association between the magnitude of codon usage bias and GC-content in third codon positions. In addition, the tRNA-gene population in *L. sinapis* showed higher GC-content at third codon positions compared to coding sequences in general and less overrepresentation of A/T-ending codons. There was an inverse relationship between synonymous substitutions and codon usage bias indicating selection on synonymous sites. We conclude that the evolutionary rate in Lepidoptera is affected by a complex interaction between underlying G/C -> A/T mutation bias and partly counteracting fixation biases, predominantly conferred by overall purifying selection, gBGC, and selection on codon usage.

**Significance**

The rate and patterns of nonsynonymous and synonymous substitutions are commonly used to distinguish between neutral evolution and selection on protein evolution. However, the extent of other forces acting on putatively neutral sites is unknown in many organisms, this could potentially affect the inference of selection or evolutionary constraints. Here, we investigate the relationship between gene sequence evolution and nucleotide composition, and the extent of mutation bias and fixation biases, such as GC-biased gene conversion (gBGC) and codon usage bias in Lepidoptera. We found that the evolutionary rates and nucleotide composition are affected by a complex interplay between purifying selection, mutation bias, gBGC, and selection on codon usage, which should be considered when evaluating estimates of selection.

## Introduction

Classification of gene categories evolving under influence of natural selection and other evolutionary forces is key for understanding the microevolutionary processes acting in natural populations. Identification of positively selected genes can inform on the importance of specific traits for local adaptation and lineage divergence (Stinchcombe and Hoekstra 2008). A common way to discriminate between natural selection and neutral evolution has been to compare information from substitutions at presumably neutral sites (synonymous sites in coding sequence, or, alternatively, sites in noncoding genomic regions) to sites where point mutations cause amino acid changes (nonsynonymous sites) (Kimura 1977; Nielsen 2005; Booker et al. 2017). However, in many nonmodel organisms, it is unknown how forces like mutation bias, and different fixation biases, like GC-biased gene conversion (gBGC) and codon usage preferences, shape patterns of substitution and consequently affect the inference of positive selection or evolutionary constraints.

GC-biased gene conversion is a fixation bias resulting from the preferential utilization of G/C over A/T nucleotides in heteroduplex DNA in the recombination-associated double-strand break repair mechanism (Marais 2003; Duret and Galtier 2009). The effect of gBGC counteracts the AT-mutation bias present in most eukaryotes (Lynch 2007), thereby maintaining or increasing the GC-content, depending on the relative strength of the two forces (Muyle et al. 2011). This fixation bias mimics directional selection and can lead to erroneous interpretation of adaptive evolution (Nagylaki 1983).

Codon usage bias is the preferential usage of particular codons for a specific amino acid, either by compositional biases or selection on specific codons (Sharp 2001). The mechanisms by which codon usage influence transcription and translation are complex and the interaction can occur on many levels including efficiency of translation, number of tRNA-gene copies, mRNA secondary structure and stability, and protein structure and integrity (Garel 1974; Duret 2000; Presnyak et al. 2015; Zhao et al. 2017; Liu 2020). The strength and direction of codon usage bias vary considerably between different organisms. For example, *Drosophila* and other Diptera mainly display GC-biased codon usage with tRNA availability as a major determinant, while Hymenoptera present an AT-biased codon usage predominantly governed by variation in nucleotide composition and weak translational selection (Moriyama and Powell 1997; Jørgensen et al. 2007; Vicario et al. 2007; Behura and Severson 2012; Dennis et al. 2020). Codon usage preferences can lead to selective constraints and a lower than expected rate of synonymous substitutions than under strict neutrality (McVean and Charlesworth 1999).

In this study, we focus on butterflies and moths (Lepidoptera). The order comprises over 175,000 species worldwide (Shields 1989) and butterflies have for long been tractable study organisms in evolution and ecology research (Boggs et al. 2003). Many lepidopterans are also of direct economic importance, for instance by acting as disease agents and pollinators and pests of agricultural crops. Previous work on lepidopteran comparative genomics has focused on identification of positively selected genes in specific lineages (Cong et al. 2015a; Iijima et al. 2018; Li et al. 2019), but these attempts have not addressed potential confounding effects of base composition (i.e., a proxy for gBGC) and codon usage bias on gene sequence evolution. The presence of a moderate fixation bias of synonymous substitutions towards an increase in GC-content has been identified in population studies in Lepidoptera (Galtier et al. 2018; Boman et al. 2021). Although the impact of gBGC appears to be lower in Lepidoptera compared to for example birds (Glémin et al. 2015; Bolívar et al. 2018; Barton and Zeng 2021), such a fixation bias in combination with codon usage preferences can have considerable effects on evolutionary rate estimates. So far, studies of codon usage preferences have been limited in Lepidoptera, and they have only covered specific genes in *Bombyx mori* and a single pair of other moth species (Frohlich and Wells 1994; Sharma et al. 2012).

Here, we used a selection of representative genome assemblies from all major butterfly families to characterize base composition and codon usage frequencies and to assess potential associations with gene sequence evolution. In addition, we investigated the core functions of the most conserved butterfly gene set. We also included population assessment of synonymous substitution bias and tRNA-gene abundance in one species, the wood white butterfly (*Leptidea sinapis*), for a more in-depth analysis of the underlying molecular processes.

## Results

**Fig. 1.**
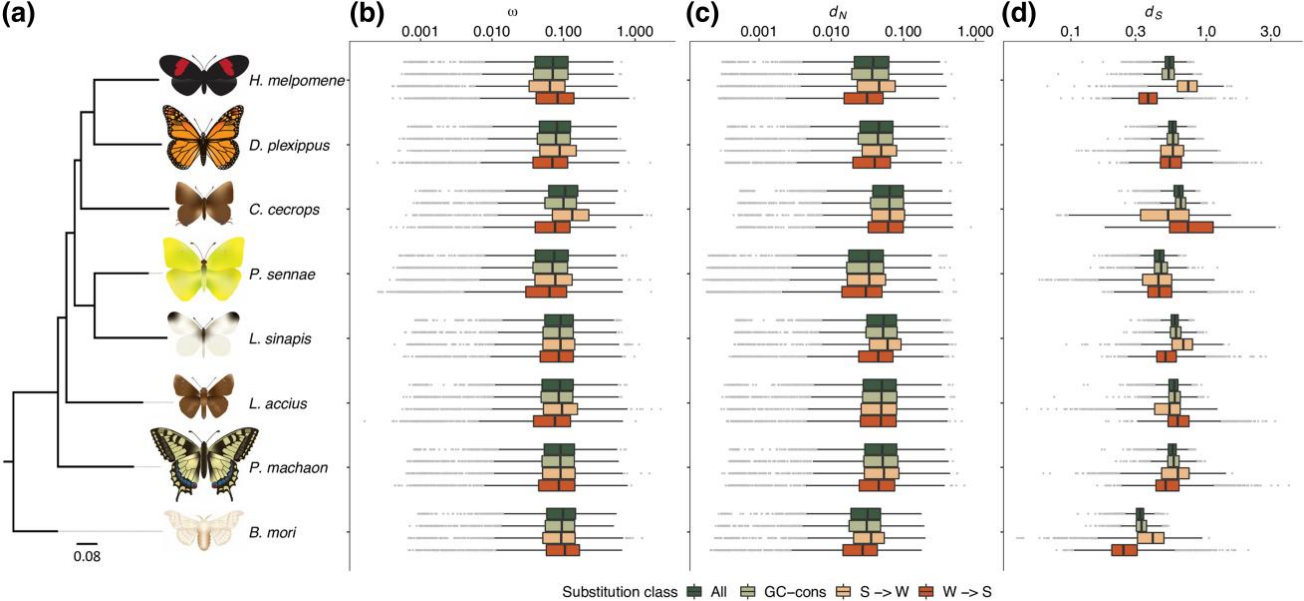
a) Phylogeny of the included taxa. Boxplots showing lineage-specific estimates of b) ω, c) nonsynonymous (*d_N_*), and d) synonymous (*d_S_*) substitution rates for all, GC-conservative (GC-cons), GC-decreasing (S -> W), and GC-increasing (W -> S) substitutions. Boxes represent first and third quartiles, the horizontal lines within boxes show median values, whiskers represent upper and lower values (1.5 * interquartile range), and the solid dots indicate outliers.

Protein coding sequences representing single copy orthologues from seven phylogenetically representative butterfly lineages, including clouded skipper (*Lerema accius*) (Cong et al. 2015a), cloudless sulphur (*Phoebis sennae*) (Cong, Shen, Warren, et al. 2016), common wood white (*Leptidea sinapis*) (Talla et al. 2017), red-banded hairstreak (*Calycopis cecrops*) (Cong, Shen, Borek, et al. 2016), monarch (*Danaus plexippus*) (Zhan et al. 2011), postman (*Heliconius melpomene*) (Davey et al. 2016), swallowtail (*Papilio machaon*) (Cong et al. 2015b), and the silkmoth (*Bombyx mori*) (Kawamoto et al. 2019), were included in the study (fig. 1, supplementary table S1, Supplementary Material online).

**Fig. 2.**
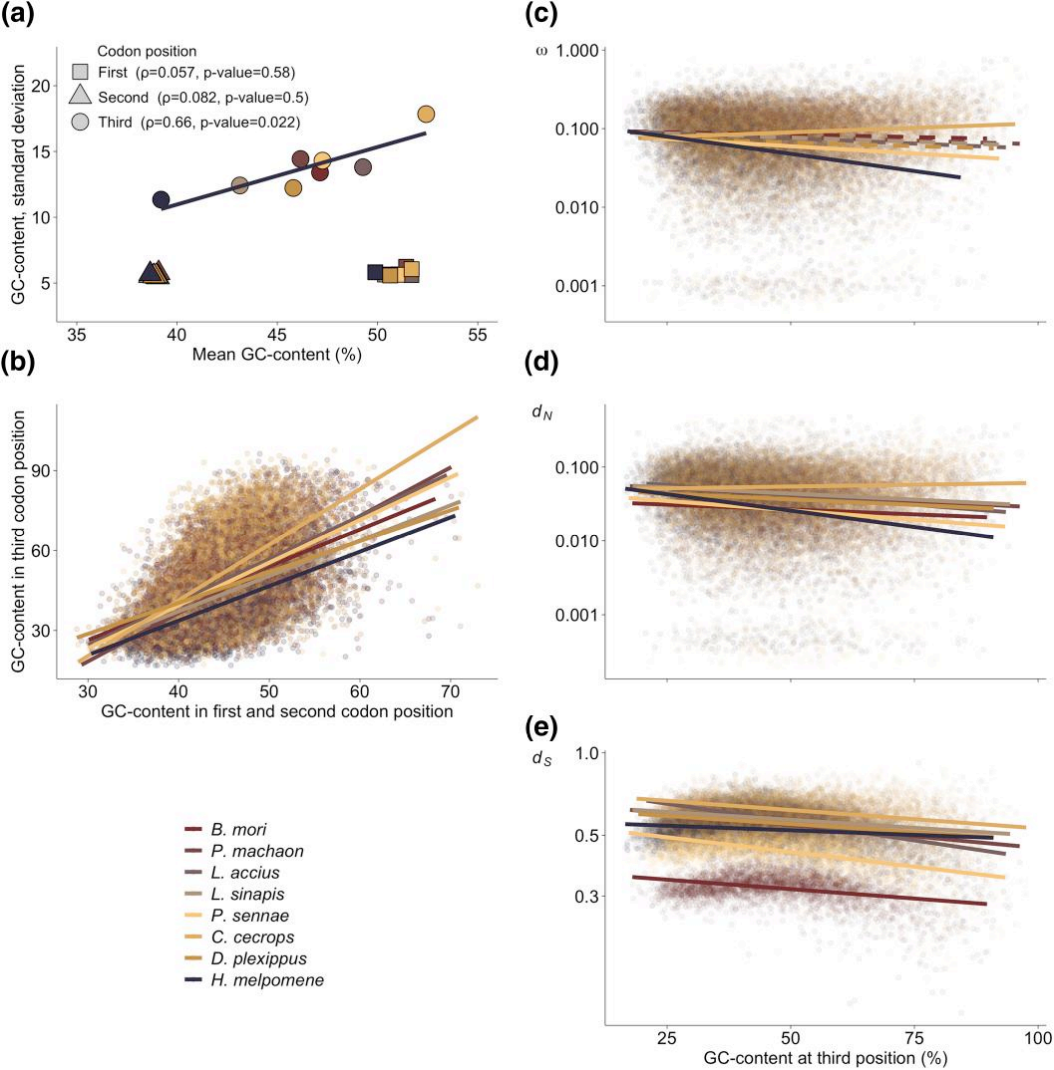
a) Relationship between standard deviation (*y* axis) and mean (*x* axis) for lineage-specific gene-wise GC-content in first, second, and third codon position. Spearman's rank correlation coefficients (*ρ*) and *P* values are shown. b) Dot plots of gene-wise GC-content in third codon position (percent, *y* axis) as a function of the mean GC-content in the first and second codon position (percent, *x* axis) per gene and branch in the aligned gene set. Lineage-specific relationship between c) ω, d) *d_N_*, and e) *d_S_* and GC-content in third codon position per gene, including all substitution types. Solid lines represent linear regressions with significant correlation coefficients, dashed lines are nonsignificant.

The fractions of coding sequences orthologous to *B. mori* for each of the investigated butterfly lineages ranged between 56% (*n* = 12,137 genes) in *H. melpomene* and 74% (11,298) in *D. plexippus*, and the number of 1:1 reciprocal orthologs across all lineages was 6,674 (30–44% of all annotated genes in a specific lineage; supplementary table S1, Supplementary Material online). After aligning and filtering, 4,150 genes remained (the aligned gene set). To estimate how representative the aligned gene set was in terms of nucleotide composition, it was compared to the entire coding DNA sequence data set (CDS) from each lineage. The total GC-content was similar between the CDS (46.6%) and the aligned gene set (45.7%; Wilcoxon's sum rank test, *W* = 44, *P* value = 0.23). The only difference was found in second codon positions where the GC-content in the aligned gene set was slightly but significantly lower (39.1%) than in the CDS (40.3%; *W* = 64, *P* value = 9.3*10^−4^). Within the aligned gene set, the mean GC-content varied significantly between first (51.2%), second (39.1%), and third codon positions (46.9%; Kruskal–Wallis test, χ^2^ = 24.78, *P* value = 1.7*10^−5^). The third codon position showed highly variable GC-content between lineages, ranging between 39.7% in *H. melpomene* and 53.1% in *C. cecrops* (fig. 2*a*, supplementary table S2, Supplementary Material online). There was a strong within-lineage association between average GC-content between first/second codon positions and third codon positions (Spearman's rank correlation coefficient; *ρ* = 0.48–0.66, *P* value < 2.2*10^−16^; fig. 2*b*). This shows that the GC-content in third codon position is not independent of the regional composition, or possibly under selective constraint. We also observed a positive relationship between the mean and standard deviation of the GC-content in third codon positions, indicating larger heterogeneity in GC-rich genomes (Pearson's product-moment correlation coefficient; *ρ* = 0.66, *P* value = 2.2*10^−2^; fig. 2*a*). The distribution of GC-content among genes within each species was relatively uniform (supplementary fig. S3*a*, Supplementary Material online). A few functional categories were significantly overrepresented in the aligned gene set compared to the CDS gene sets. The highest enrichment score was found for genes associated with 1) regulation of stress-activated MAPK cascade, regulation of mitotic events, and ribosomal subunit assembly (biological function), 2) replication recognition complex, endosome, and transcription factor complex (cellular component), and 3) DNA helicase activity, ribosome structure, and DNA replication (molecular function) (supplementary table S3, Supplementary Material online). The estimated ω across branches (Model M0) was <0.2 for all genes in the aligned gene set (supplementary fig. S1, Supplementary Material online), demonstrating that this set of genes is considerably conserved across the butterfly taxa included in the analysis.

Next, we compared the relationship between sequence conservation and gene characteristics by comparing the 100 genes with the lowest ω to the rest of the aligned gene set. There was no difference in overall or third codon position GC-content between the gene sets, although there was a small difference in mean GC-content for first (52.5 ± 5.5% vs. 51.2 ± 5.6%, *W* = 0, *P* value = 1.6*10^−4^) and second codon positions (37.5 ± 5.5% vs. 39.1 ± 5.5%; *W* = 64, *P* value = 1.6*10^−4^). Significantly overrepresented ontology terms in the conserved gene set were associated with housekeeping functions like proteasome complex, cytosolic small ribosomal subunit, spliceosomal complex, and Sm-like protein family complex (cellular component), and structural constituent of ribosome and structural molecule activity (molecular function) (supplementary table S4, Supplementary Material online). In addition, we performed a branch-site test for positive selection using the aligned gene set and *L. sinapis* as the foreground branch and found 41 candidate genes under positive selection. A gene ontology analysis did not reveal any enrichment for specific functional categories in the positively selected genes, but the mean GC-content in third codon positions was higher than in the aligned gene set in general (see supplementary analysis, Supplementary Material online).

### Lineage-specific Evolutionary Rate Variation in Lepidoptera

We applied two different methods to estimate the substitution rates; *codeml* as implemented in PAML for comparisons between lineages and *mapNH* in the Bio++ suite for analyzing associations with nucleotide composition and codon usage. The two methods gave different lineage-specific estimates of ω, likely due to differences in the underlying models for inferring the substitutions rates, but the relative differences between lineages were similar (supplementary tables S5 and S6, Supplementary Material online). Mean ω ranged from 3.5*10^−2^ (*P. sennae*) to 4.8*10^−2^ (*L. sinapis*) for the *codeml* branch model and from 8.4*10^−2^ (*H. melpomene*) to 1.2*10^−1^ (*C. cecrops*) when *mapNH* was applied. We applied a generalized linear mixed model analysis with *B. mori* as reference and found a significant difference in ω between lineages (glmer; intercept 0.03, marginal *R*^2^ = 0.017, supplementary table S6, Supplementary Material online). This analysis also showed that the lineages can be grouped in three distinct categories with similar within-group estimates of ω; 1) *H. melpomene*, *D. plexippus*, *P. sennae* (lowest ω; estimates 0.80–0.88, *P* values < 1.00*10^−04^), *L. accius* and *P. machaon* (intermediate ω, estimates 1.00–1.01, not significantly different from *B. mori*), and 3) *C. cecrops* and *L. sinapis* (highest ω; estimates 1.11–1.16, *P* values < 1.00*10^−04^; fig. 1*b*, supplementary fig. S2, Supplementary Material online, supplementary table S6, Supplementary Material online).

### Evolutionary Rates for Different Substitution Classes

To further characterize the different types of substitutions and potential rate variation associated with base composition, the evolutionary rates of W -> W and S -> S (GC-conservative), W -> S (GC-increasing), and S -> W (GC-decreasing) substitutions were estimated across the different lineages. We found that ω was significantly higher in all lineages, except *H. melpomene*, when using the global branch model (all types of substitutions combined) compared to using the GC-conservative substitutions only (paired Wilcoxon signed rank test, *P* values = 9.50*10^−02^ – 2.20*10^−16^, fig. 1, supplementary table S7, Supplementary Material online). The differences observed in most of the lineages are likely an effect of higher GC-conservative synonymous substitution rate, while the differences in nonsynonymous substitution rates were negligible with the exception of *H. melpomene* and *P. machaon*. To further analyze how the directions of substitutions were influencing the difference between global and GC-conservative estimates, we looked at the GC-altering substitutions. Here, we found that ω was significantly higher for S -> W compared to W -> S substitutions, except in *H. melpomene* and *B. mori* where the relationship between the substitution types were reversed (paired Wilcoxon signed rank test, *P* values = 9.50*10^−02^ – 2.20*10^−16^, fig. 1, supplementary table S7, Supplementary Material online). Although the nonsynonymous substitutions showed a consistently higher rate of GC-decreasing substitutions in all lineages, they were counteracted by the W -> S substitution rates being consistently lower thereby having a minor impact on the difference in ω between global and GC-conservative estimates. The synonymous rates were considerably more variable between categories and lineages. The differences between the substitution types were significant in all species, with the most remarkable differences in *C. cecrops* which had a substantially higher W -> S substitution rate and *H. melpomene* with a higher S -> W substitution rate (fig. 1, supplementary table S7, Supplementary Material online). The main direction of the synonymous substitution rate was variable between lineages, but for most of the lineages the sum of the impact resulted in a reduction in the global rate compared to GC-conservative rates.

In summary, we observed a higher ω for global substitutions compared to GC-conservative substitutions in most of the species, as a result of the higher S -> W than W -> S evolutionary rate observed in the majority of the analyzed species. However, the underlying substitution rates, predominantly the synonymous rate, showed a lineage-specific and highly variable main direction of change.

### Effects of Nucleotide Composition on Substitution Rate Estimates

To assess the effects of nucleotide composition on evolutionary rate estimates, lineage-specific correlations between GC-content and substitution rates were performed for each substitution class. Significant negative associations were observed between GC-content and ω for GC-conservative, W -> S and the global set of substitutions in all lineages, except in *C. cecrops* (Spearman's rank correlation, supplementary table S8, Supplementary Material online, fig. 2). For S -> W substitutions, there was a positive association between GC-content and ω in all species except *H. melpomene* (supplementary table S8, Supplementary Material online, supplementary fig. S3, Supplementary Material online). The relationship between GC-content and *d_N_* and *d_S_* was similar to ω for GC-conservative and global substitutions, but *d_S_* had a strong negative association to S -> W and positive association to W -> S substitutions (supplementary table S8, Supplementary Material online, supplementary fig. S3, Supplementary Material online). To visualize the association between GC-content and ω, we applied a local regression model which revealed a u-shaped relationship between lineage-specific ω and GC-content in third codon positions. This indicates higher ω for genes with particularly low or particularly high GC-content (although the variance also increased noticeably at the tails of the distribution; supplementary fig. S3, Supplementary Material online). The associations between GC-content and ω estimates, exacerbated in *H. melpomene* and *C. cecrops,* indicate that inferences of evolutionary rates in Lepidoptera are dependent on lineage-specific differences in the mutation bias, and potential nonselective fixation biases (e.g., gBGC).

### Evidence for Fixation Bias Affecting Synonymous Substitutions

**Fig. 3.**
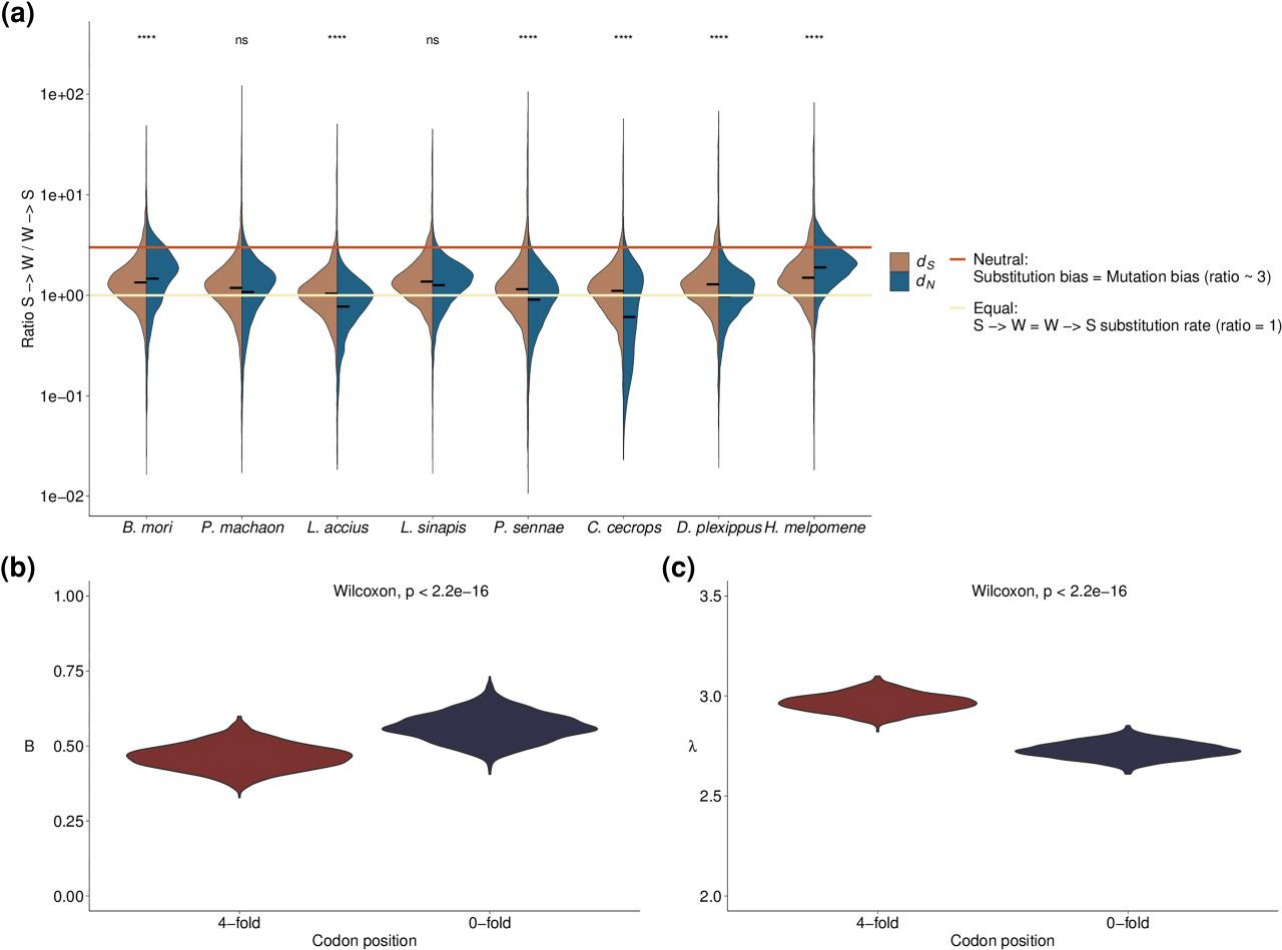
a) Distribution and mean (horizontal bar) for S -> W/W -> S substitution rate ratios for synonymous (*d_S_*, left distribution) and nonsynonymous substitution rates (*d_N_*, right distribution). The horizontal lines represent the expected S -> W/W -> S ratios if the rates for the two substitution categories would be identical to each other (1:1, ratio = 1) or exactly reflect the expected mutation bias (3:1, Ratio ≈ 3). Distribution of population estimates of b) fixation bias favoring GC (B, *y* axis) and c) mutation bias (λ, *y* axis), on different codon positions (*x* axis) in *Leptidea sinapis*, after bootstrapping (1,000 samplings with replacement).

GC-content is determined by the counteracting forces of mutation bias and fixation biases such as GC-biased gene conversion and selection. We can infer the determinants of GC-content evolution by evaluating the S -> W/W -> S substitution rate ratio. Assuming a neutral evolutionary history for synonymous substitutions, we would expect a S -> W/W -> S substitution rate ratio close to the mutation bias, which in Lepidoptera has been estimated to be approximately 2.6–3.3, dependent on the local GC-content (Boman et al. 2021). Using population resequencing data from 10 *L. sinapis* individuals and a population genetics model taking both GC-content and gBGC into account (Glémin et al. 2015), we estimated the average S -> W/W -> S mutations bias at codons to 2.96 at 4-fold and 2.73 at 0-fold degenerate codon positions (fig. 3*c*). However, the mean and standard deviation of synonymous S -> W/W -> S substitution rate ratio across lineages was 1.34 ± 0.89 (median = 1.19), similar to the ratio for nonsynonymous substitutions (mean = 1.89 ± 3.89; median = 1.23), which presumably includes a substantial fraction of positions under purifying selection (fig. 3*a*, supplementary table S9, Supplementary Material online).

The discrepancy between the estimates of S -> W/W -> S mutation- and substitution biases is not expected if synonymous substitutions only experience neutral evolution. This indicates that the fixation probabilities of a considerable subset of synonymous mutations are affected by fixation biases (e.g., gBGC and selection for specific codons) that should be considered when estimating evolutionary rates in Lepidoptera. With the same population genetics model as above (Glémin et al. 2015), we estimated that the fixation bias favoring a higher GC-content (B) for 4-fold degenerate codon positions in *L. sinapis* to be 0.46. Although this is lower than the estimates for 0-fold degenerate codon positions (B = 0.56), this points to considerable deviations from neutrality at synonymous sites in the wood white (fig. 3*b*).

### Codon Usage Within Lepidoptera is Mainly a/T-biased

To characterize codon usage frequencies in Lepidoptera and assess potential effects on base composition and substitution patterns, we calculated the frequency of each of the 61 different codons that encode amino acids (ignoring the three stop codons) per 1,000 codons for each species and compared codon frequencies between the different gene sets (supplementary table S10, Supplementary Material online). The codon usage frequencies in the aligned gene set were representative for the overall CDS gene set (paired Wilcoxon's signed rank test, *W* = 55,849, *P* value 0.22). To investigate the association between nucleotide composition in third codon positions and lineage-specific synonymous codon usage in the aligned gene set, we used a measure of relative codon usage (RSCU), where 1 means equal usage of all synonymous codons, >1 means increased relative usage and <1 means decreased relative usage. All lineages except *C. cecrops* showed a higher proportion of A/T-ending codons with RSCU > 1 (supplementary fig. S4, Supplementary Material online, supplementary table S11, Supplementary Material online).

### Copy Number Distribution and GC-content in tRNA genes

**Fig. 4.**
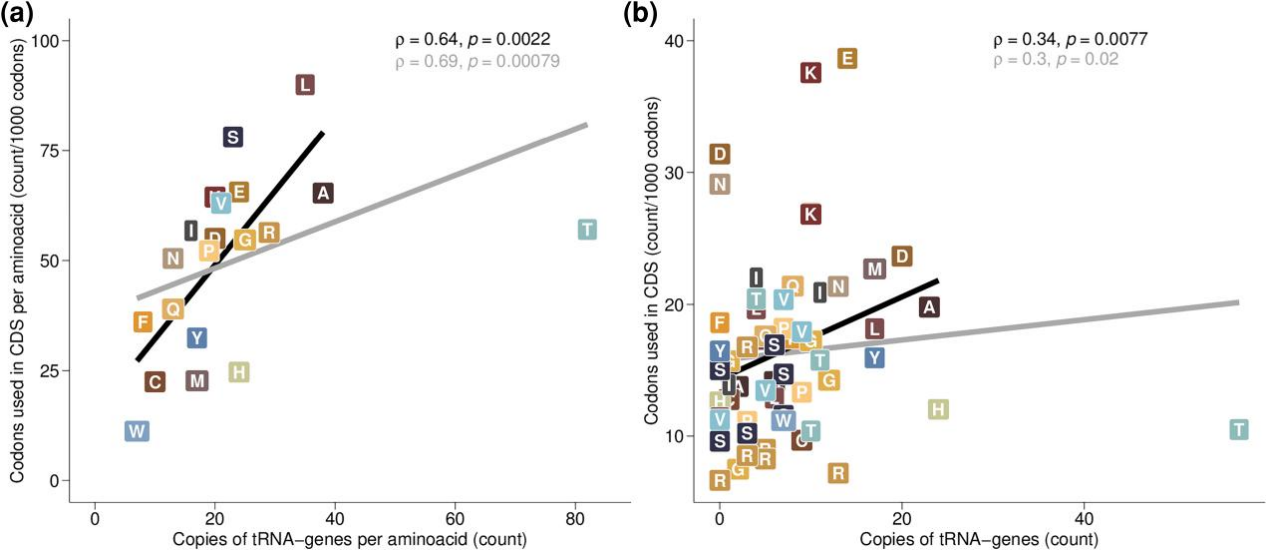
a) Frequency of codons per amino acid in *Leptidea sinapis* as a function of the number of copies of tRNA genes per amino acid. b) Frequency of codons used in *L. sinapis* as a function of the number of copies of tRNA genes per codon. The squares are colored by amino acid, and letters represent the standard amino acid symbol. The lines represent linear regressions of all codons (light) and codons excluding T^ACC^ (dark). Spearman's rank correlation coefficients (*ρ*) and *P* values for each comparison is presented.

The observed A/T bias in codon usage and the counteracting strength of gBGC is not enough to explain the observed GC fixation bias at 4-fold degenerate sites. Selection for translational efficiency could increase the usage of codons with high tRNA abundance or vice versa; maintenance of high number of tRNA-gene copies with anticodons corresponding to commonly used codons could potentially increase translation efficiency. We analyzed the copy number variation of tRNA genes in the newly assembled chromosome-level *L. sinapis-*genome as a proxy for tRNA abundance. The total number of predicted functional tRNA genes was 461, with 50 unique anticodons of 61 possible anticodons (supplementary table S12, Supplementary Material online). The median copy number of represented anticodons was 7 (range 1–24) with threonine (T^ACC^) as an outlier having 57 copies (supplementary table S12, Supplementary Material online). It is possible that the presence of an exceptionally large number of copies of potentially functional T^ACC^ is a consequence of recent repeat proliferations (e.g., many SINEs contain tRNA-gene copies) and that the software cannot distinguish these from “true” tRNA-gene models. The distribution of codons in the tRNA-gene set deviated significantly from random usage of the synonymous codons (Pearson's χ^2^ test, X^2^ = 165.11, df = 60, *P* value = 9.34*10^−12^), this was significant also when excluding the outlier codon T^ACC^. There was a strong association between the counts of amino acids in the CDS and number of tRNA-gene copies for each amino acid (Spearmans's rank correlation coefficient *ρ* = 0.64, *P* value 2.2*10^−03^; fig. 4*a*), the correlation was significant also when excluding T^ACC^ (*ρ* = 0.69, *P* value 7.9*10^−04^).

There was a slightly higher frequency of codons in the CDS with a corresponding specific tRNA-anticodon compared to unrepresented codons, but this difference was not significant (Wilcoxon rank sum test, W = 309, *P* value = 3.92*10^−1^). We also found an association between the counts of used codons and the copy number of tRNA genes for each anticodon (*ρ* = 0.3, *P* value 2.0*10^−2^; fig. 4*b*). This association could be partly driven by the strong covariation in the amino acid counts, because there is no significant association between the ratio of usage of specific anticodon within each tRNA isoacceptor family and synonymous usage of specific codons in the CDS (i.e., RAIT vs. RSCU; *ρ* = 0.18, *P* value = 0.753). For overrepresented tRNA-species (RAIT > 1), there was 12 A/T and 10 G/C ending tRNA codons, which was less A/T-biased than the overrepresented codons in the CDS in *L. sinapis* (25 A/T, 4 G/C). The GC-content among the tRNA genes at third codon positions was 57.9% (52.0% excluding T^ACC^), which is higher than for third codon positions in the CDS gene set (44.7%; supplementary table S12, Supplementary Material online). In summary, there was an association between codon usage and the distribution of tRNA genes, but the A/T codon usage bias in third codon positions was not represented in the tRNA-gene set. The third codon positions in tRNA genes had a higher GC-content in total and a lower A/T bias, which potentially could influence codon usage and contribute to the fixation bias increasing GC-content in third codon positions.

### The Effective Number of Codons Vary Among Lineages

**Fig. 5.**
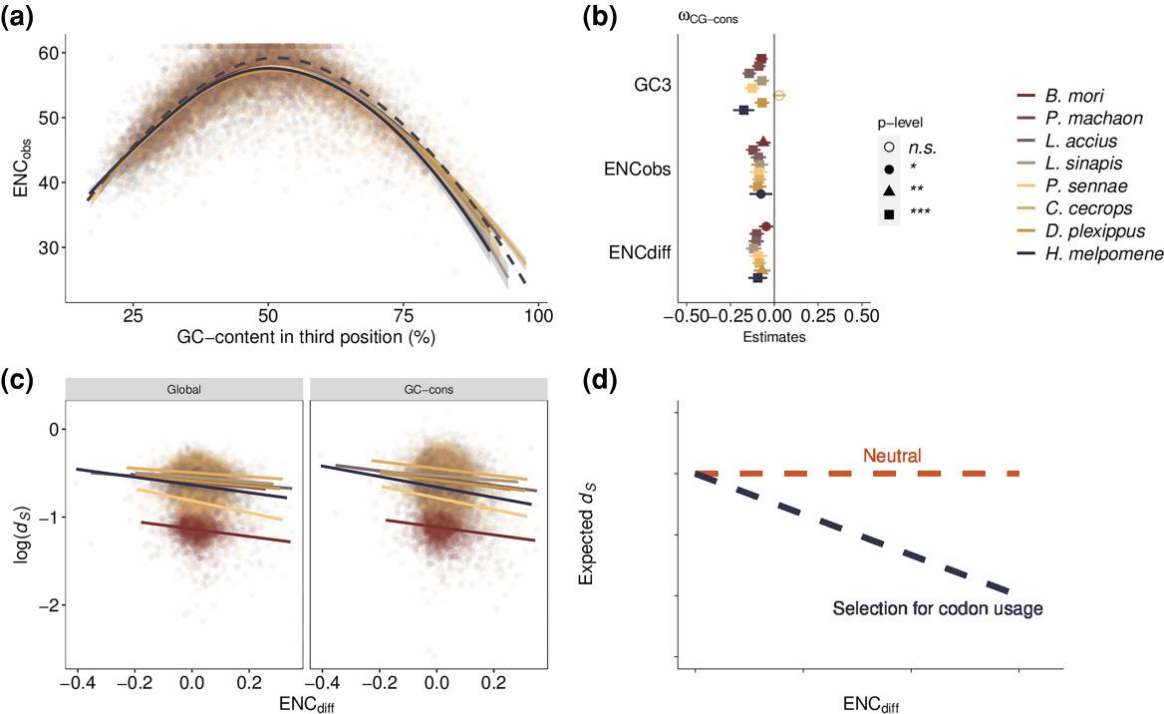
a) Lineage-specific observed effective number of codons per gene (ENC_obs_; dot plot, color by lineage), local regression (LOESS) models for observed ENC (solid lines), and expected ENC (dashed lines) as a function of GC-content in third codon positions. b) Linear regression estimates for each lineage with GC-conservative estimates of ω as a function of GC-content in third position, observed ENC and difference between expected ENC and observed ENC (ENC_diff_). c) Lineage-specific synonymous substitutions (*d_S_*) for each gene as a function of the difference between expected and observed effective number of codons (ENCdiff). Lines represent linear regression model for each branch. d) Cartoon of expected relationship between *d_S_* and ENC_diff_ if the synonymous substitutions are neutral or if selection on codon usage is affecting the fixation rate of synonymous mutations.

To further investigate how codon usage affects the substitution rate, we estimated the effective number of codons (ENC) score. The ENC score can range from 20 (complete codon usage bias) to 61 (all codons are used to the same extent), and we found that the mean estimates of ENC-scores across genes ranged between 51.9 (*C. cecrops*) and 54.3 (*L. accius*) (range for individual genes: 21–61; median = 54.3; fig. 5*a*, supplementary table S13, Supplementary Material online). The observed ENC-scores were positively associated with the GC-content in all lineages, except in *C. cecrops* where the correlation was negative (Spearman's rank correlation, *ρ* =−0.17–0.70, *P* value < 2.2*10^−16^; supplementary fig. S5, Supplementary Material online).

### Codon Usage Bias Contributes to Differences in Substitution Rates Among Genes and Lineages

If the estimates of ω are inflated by purifying selection by codon usage preferences at third codon positions, we would expect a lower synonymous substitution rate where codon usage bias is stronger, under the assumption that codon usage bias is at equilibrium. To get an estimate of the magnitude of codon usage bias while controlling for GC-content, we compared the observed and expected ENC-scores based on overall nucleotide composition in each lineage (ENC_diff_). The lineage-specific ENC_diff_ were on average 0.016–0.025, with a larger bias in genes with higher GC-content (Spearmans's rank correlation coefficient, *ρ* = 0.035–0.13, *P* value 5.8*10^−02^–5.1*10^−12^; supplementary fig. S5, Supplementary Material online), in contrast to the observed overrepresentation of A/T-ending codons in most of the lineages. There were significant negative associations between ENC_diff_ and *d_S_* (*ρ* = −0.05 to −0.19, *P* value 2.2*10^−16^ – 4.0*10^−03^; fig. 5*c*, supplementary table S13*c*, Supplementary Material online), in all species except *D. plexippus*, and the negative relationship remained when only GC-conservative substitutions were included. This again indicates selection on synonymous sites and that codon usage preferences influence substitution rate estimates. We found a trend towards negative associations between ENC_diff_ and GC-conservative estimates of ω in the majority of lineages (*ρ* = 0.01–0.07, *P* values = 3.7*10^−04^ – 9.5*10^−01^; supplementary table S13*c*, Supplementary Material online). In addition, when binning the most conserved genes and comparing with the rest of the aligned gene set, we observed a trend towards stronger codon usage bias in the conserved gene set. The stronger codon usage bias in more conserved genes was consistent across all lineages, but not significant for all (Wilcoxon's rank sum test; *P* values = 1.9*10^−05^ – 8.7*10^−01^; supplementary fig. S5, Supplementary Material online). Taken together, these results show that codon usage bias contributes to differences in substitution rates and could lead to erroneous inference of the mode of natural selection, especially at the level of individual genes.

### Synthesizing the Effects of Base Composition and Codon Usage Bias on ω

In order to assess the relative effects of different factors on the overall evolutionary rate in Lepidoptera, we applied a linear model with ω as the dependent variable and ENC and ENC_diff_, together with GC-content in third codon positions as explanatory variables. The variance in ω was partly explained by all three variables in varying degrees among the lineages (linear regression, F = 11.60–47.46, *P* value < 2.2*10^−16^; fig. 5*b*, supplementary table S14, Supplementary Material online), but the total variance in ω explained by the model was low (adjusted *R*^2^ = 0.011–0.046; supplementary table S14, Supplementary Material online). The influence of codon usage bias and GC-content on the evolutionary rate estimates shows the importance of assessing both codon frequencies and potential mutation- and fixation-biases when estimating rates and patterns of gene sequence evolution in Lepidoptera.

## Discussion

Here, we explored the impact of base composition and codon usage on evolutionary rates in Lepidoptera. It should be noted that the particular taxonomic sample set used for analysis obviously affects the potential to study microevolutionary processes, since the expected level of conservation depends both on the number of lineages and the variance in divergence times between sampled taxa. The set of genes identified as 1:1 orthologs across all Lepidoptera lineages (the aligned gene set) evidently includes the most conserved genes in the clade. The low overall evolutionary rate estimates for this gene set supports considerable effects of purifying selection. As expected, given the inherent bias towards conserved genes in the aligned gene set, we found that the overrepresented functional categories were associated with core cellular processes like translational activity and DNA maintenance (Stein et al. 2003). This was especially evident in the bin with the lowest ω (the conserved gene set) with overrepresentation of functions associated with pathways involved in basal cellular functions like cytoplasmic translation and proteasome function.

### Lineage-specific Rates of Evolution in Lepidoptera

The lineage-specific analyses revealed three distinct groups with significantly different ω estimates. The lowest ω was found in *H. melpomene*, *D. plexippus*, and *P. sennae*, while *B. mori*, *L. accius*, and *P. machaon* showed intermediate ω and *C. cecrops* and *L. sinapis* the highest ω. Differences in demographic histories between the lineages could affect the evolutionary rates, as variation in effective population size (*N_e_*) determines the efficacy of selection, thereby predominantly influencing the accumulation of slightly deleterious nonsynonymous mutations in lineages with lower *N_e_* (Galtier 2016). It should be noted that both *L. sinapis* and *C. cecrops* have larger genomes than the other species in the data set, predominantly a consequence of accumulation of TEs (Cong, Shen, Borek, et al. 2016; Talla et al. 2017). In *L. sinapis*, the level of heterozygosity is also comparatively low (Mackintosh et al. 2019; Talla et al. 2019). Hence, a comparatively low long-term *N_e_* in *L. sinapis* may have resulted in both accumulation of repeats and slightly deleterious mutations, explaining the higher estimate of ω in this lineage.

An alternative, but not mutually exclusive explanation for the observed phylogenetically disordinate differences in evolutionary rate could be differences in generation time. Some species, in particular in temperate regions, enter diapause after a single life cycle, while other species have several (up to ≈10) complete life cycles per breeding season (Tolman and Lewington 2009), thereby accumulating more mutations due to an increased per time unit rate of DNA replication errors (Nabholz et al. 2007; Thomas et al. 2010). Such an effect should mostly be at play for synonymous substitutions (but see e.g., Thomas et al. 2010) while the effect on nonsynonymous sites is more complex. An increased nonsynonymous mutation rate as a consequence of short generation time could be canceled out by more efficient selection against slightly deleterious variants, since species with shorter generation times tend to have larger *N_e_*, although we do not know if this is the case for the species included in our data set (Ohta 1992). No associations between nonsynonymous substitution rates and body size (Thomas et al. 2006) or metabolic rate (Lanfear et al. 2007) have been observed in Lepidoptera, traits that often are used as proxies for *N_e_*. Furthermore, levels of genetic diversity in butterflies are generally negatively correlated with body size, but not with other life history traits (Mackintosh et al. 2019). Further studies including more taxa should give more information on the relationship between gene sequence evolution and life history traits.

### Effects of Base Composition, gBGC, and Codon Usage on Evolutionary Rates

Our analyses showed a higher ω for global substitutions compared to GC-conservative substitutions in most of the species, indicating that the nucleotide composition influence the inference of evolutionary rates. The nucleotide composition was highly variable between lineages, especially for the third codon positions. We also found a strong association between GC-content at first and second codon positions, which generally can be assumed to be under strong purifying selection, and the GC-content at third codon positions where most sites are synonymous. Potential evolutionary forces that may contribute to the observed patterns include mutation bias (Petrov and Hartl 1999; Keightley et al. 2015; Boman et al. 2021), gBGC, and selection on codon usage (Marais 2003; Martin et al. 2016). It is well established that multicellular eukaryotes in general experience a S -> W mutation bias (Long et al. 2018), predominantly as a consequence of spontaneous deamination. Such a mutation bias has also been observed in several *Leptidea* species and seems to be pervasive in butterflies which in general have low genome-wide GC-content (0.3–0.4) (Challis et al. 2016; Boman et al. 2021). We observed higher rates of S -> W than W -> S substitutions at both nonsynonymous and synonymous sites in the majority of the investigated lineages, supporting a general effect of the S -> W mutation bias on gene sequence evolution in butterflies. The general negative association between substitution rates and GC-content are also in line with a higher number of random fixations of S -> W mutations. However, the ratio of S -> W over W -> S synonymous substitutions was lower than expected from the mutation bias alone in the majority of species. Both the difference between the observed and expected ratios of S -> W/W -> S substitutions and the presence of a GC-favoring fixation bias at third codon positions are in line with an effect of gBGC, the GC-biased repair of double-strand breaks during homologous recombination observed in many taxa across the tree of life (Marais 2003; Duret and Galtier 2009). GC-biased gene conversion mimics directional (positive) selection as the efficiency of the process depends on the recombination rate and *N_e_*. This means that the impact of gBGC on sequence evolution should vary across lineages that differ in demographic history and recombination rate (Glémin et al. 2015). We found that nucleotide composition varied considerably between butterfly species, and there was a higher heterogeneity in regional nucleotide composition in species with a higher GC-content. This indicates that the intensity of gBGC has varied between species and likely also between genomic regions as a consequence of differences in the recombination landscape (Figuet et al. 2015). If gBGC had been a dominating force in Lepidoptera, the expectation would be that W -> S substitutions should constitute a large proportion of the total number of substitutions, especially at synonymous sites (Mugal et al. 2015; Bolívar et al. 2019). In contrast, we observed that S -> W substitutions contributed slightly more to the global rates, suggesting that gBGC, although its effect is substantial, only partly compensate for the strong S -> W mutation bias in some lineages. This shows that specific effects of gBGC should be investigated in more detail in Lepidoptera since it can affect the assessment of selective and neutral processes, for example, by development of detailed recombination maps and/or direct quantification of the fixation bias (Glémin et al. 2015; Kawakami et al. 2019). Our estimates of B in the coding sequences in *L. sinapis* were higher (0.46) compared to previous genome-wide estimates (≈0.2) (Boman et al. 2021), although lower compared to other Lepidoptera ranging between 0.7 and 1.16 (Galtier et al. 2018) The impact of gBGC will fluctuate with regional variation in both nucleotide composition and recombination rate, and we know from previous analyses in butterflies that the recombination rate is reduced in coding compared to intergenic sequences (i Torres et al. 2022; Shipilina et al. 2022). Hence, we would expect that the effect of gBGC should be higher in noncoding compared to coding sequences. The observed fixation bias towards increased GC-content at synonymous sites compared to genome-wide estimates therefore indicates that additional evolutionary forces, such as selection for GC-ending codons, influence the nucleotide composition at synonymous sites.

The strength and direction of codon usage bias has been shown to vary between different organisms. Parasitoid wasps have been shown to have AT-rich genomes and A/T-biased codon usage (Dennis et al. 2020), while *D. melanogaster* has high GC-content in coding sequences and a preference for GC-rich codons (Vicario et al. 2007). We found a bias towards A/T-ending codons, but stronger codon usage bias in G/C-rich genes in general. An exception was *C. cecrops,* the species with the highest average GC-content, where we observed a negative correlation between ENC_obs_ and GC-content, in line with observations in other lepidopteran species with GC-rich genomes (Sharma et al. 2012). The pattern of codon usage in Lepidoptera is hence similar to ants and honey bees, where a bias for AT-rich codons seems to be dominating (Behura and Severson 2012). We suggest that codon usage in butterflies is partly dependent on compositional constraints, as has previously been shown in for example prokaryotes, plants, humans, and flatworms (Knight et al. 2001; Palidwor et al. 2010; Lamolle et al. 2019). Nucleotide composition, however, does not fully explain the stronger codon usage bias in GC-rich genes observed in our data. We observed a strong relationship between copy number of tRNA isoacceptor genes and amino acid usage in the CDS of *L. sinapis*, similar to what has been observed for example in bacteria, mosquitos, and other organisms (Behura and Severson 2011), supporting coevolution between tRNA genes and codon usage mainly driven by amino acid usage (Lobry and Gautier 1994; Higgs and Ran 2008). However, the abundance of specific tRNA:s in the cytoplasm has been shown to be highly correlated to codon usage and a limiting factor in the translation rate of highly expressed genes (Ikemura 1982; Varenne et al. 1984). In line with this, tRNA availability is an important determinant of codon usage bias in *Drosophila* (Moriyama and Powell 1997). We found only a weak association between the synonymous codon usage and specific anticodons within each isoacceptor family, but the observed tRNA genes had considerably higher GC-content in third codon positions without the overrepresentation of AT-ending codons observed in the coding sequences. One explanation could be that the relationship between anticodons and codons are complicated by the wobble-function of the third position nucleotide, which gives one anticodon the possibility to match several nucleotides at third codon position. In addition, although tRNA-gene copy number in several organisms is proportional to the abundance of mature tRNA in the cytoplasm, it is not a direct measure (Percudani et al. 1997; Kanaya et al. 1999). The tRNA abundance in the cytoplasm could vary due to regulation of expression levels and post-transcriptional modifications depending on for example developmental stage (Moriyama and Powell 1997). Another explanation could be that there is a mismatch between the “optimal” codons for translation as determined by the tRNA-gene set on the one hand and the bias towards AT-ending codons caused by the general mutation bias on the other. The high GC-content in tRNAs could lead to an increase of the fixation of A/T -> G/C mutations as those potentially are beneficial for rapid or more accurate translation and thereby counteract the A/T accumulation caused by the mutation bias. Thus, it is possible that both the tRNA-gene set and gBGC could contribute to the observed fixation bias that leads to an increase in the GC-content in general and to the stronger codon usage bias in GC-rich genes.

Depending on the mechanism underlying the codon usage bias, we would expect different patterns on the synonymous substitution rate. If the codon usage bias is a consequence of the mutation bias alone, there would be no correlation with the GC-conservative synonymous substitution rate. If, on the other hand, the codon usage bias is an effect of selection for utilization of specific codons, the synonymous substitution rate would have a negative relationship with strength of codon usage bias, as seen in *Drosophila* (Sharp and Li 1989; Maside et al. 2004). We therefore investigated the associations between codon usage, base composition, and substitution rates and found a negative association between the strength of codon usage bias and the synonymous substitution rate. One potential caveat here is that the substitution estimates are based on a modified version of mutation opportunity when counting the proportion of synonymous sites (*MapNH*). There is hence a risk of underestimating the number of synonymous sites (the denominator in *d_S_*), which would lead to underestimation of the strength of codon usage bias (Bierne and Eyre-Walker 2003). Our data point to a significant codon usage bias in Lepidoptera in general, but these estimates are likely conservative and forthcoming efforts to quantify codon usage bias could preferably apply analysis including physical sites models. Finally, we also observed a trend towards stronger codon usage bias in more conserved genes, corroborating other studies (Rao et al. 2011), which points to potential effects of selection for translation efficiency and/or structural stability (Zhou et al. 2016; Novoa et al. 2019).

## Conclusion

Here, we characterized the relationships between gene sequence evolution and nucleotide composition in eight Lepidoptera lineages. Our analyses show that mutation-, fixation-, and codon usage biases have been key in shaping gene sequence evolution in butterflies in general. We conclude that substitution rates in lepidopteran genes are affected not only by directional selection on nonsynonymous sites, but a complex interplay between a general S -> W mutation bias and the general preference for utilization of AT-ending codons, counteracted by a significant W -> S fixation bias, likely acting in concert with selection for translational efficiency.

## Materials and Methods

### Data Collection

Protein codon sequences from seven of the taxa were downloaded from the database LepBase, http://ensembl.lepbase.org/ (Challis et al. 2016) (supplementary table S1, Supplementary Material online). The protein-coding sequences from *L. sinapis* were obtained from a previous study (Talla et al. 2017). One-to-one orthologous genes between each respective butterfly lineage and *B. mori* were identified using the *BLASTP* tool (Altschul et al. 1990). Pairwise alignments for all orthologs were generated based on translated protein sequences using *Prank* v.170427 (Löytynoja 2014). To omit potential pseudogenes, alignments that contained interspersed stop codons and/or indels that were not multiples of three, were discarded from the data set. To eliminate inadequately aligned genes, a set of filtering steps was applied. First, *Trimal* v.1.2 (Capella-Gutierrez et al. 2009) was used to remove codons with more than 50% gaps across lineages, alignments with more than 50% missing codons in any lineage and alignments with on average >25% gaps per sequence. Second, alignments shorter than 300 bp were omitted to avoid including fragmented genes. The resulting total set of alignments, from now on referred to as “the aligned gene set”, contained 4,150 genes. To get the necessary tree topology of the species’ for downstream analysis, a phylogenetic tree was constructed with a random set of 1,091 concatenated genes using *RaxML* with the GTR-gamma substitution model (Stamatakis 2014). The topology of the resulting tree was in accordance with the well-established phylogeny of the species set (Espeland et al. 2018; Kawahara et al. 2019).

### Evolutionary Rate Analysis

Mean pairwise nonsynonymous (*d_N_*) and synonymous (*d_S_*) substitution rates (number of substitutions per site category) and the *d_N_*/*d_S_*-ratio (ω) were estimated for all individual genes in all Lepidoptera lineages using model M0 in *codeml*, as implemented in *PAML* v.4.9e (Yang 2007). Alignments were excluded if gene wide *d_S_* > 30 (saturation) or if ω ≥ 999 (absence of synonymous substitutions in the sequence). Evolutionary rate variation among lineages was estimated by applying the free-ratio branch model, as implemented in *codeml* in *PAML* v. 4.9e (Yang 2007). To assess potential differences in ω between lineages, we applied a generalized linear mixed model with ω as dependent variable, branch as a fixed variable and gene identity as a random variables, using the *glmer* function as implemented in *lme4* (version 1.1–28); glmer(dnds∼branch+(1|GeneID), data = branch_paml_long, family = Gamma(link = “log”)) (Bates et al. 2015). In addition, we identified candidate genes under positive selection in the aligned gene set in *L. sinapis* using *codeml* (see supplementary analysis, Supplementary material online).

To investigate the impact of nucleotide composition on the evolutionary rate estimates, we also applied a model inferring the rates of different substitution classes. The substitutions were categorized according to the bases involved and their effect on base composition. Since G–C base pairs have three hydrogen bonds, they are referred to as strong (S), while A–T base pairs that only have two hydrogen bonds are referred to as weak (W). Hence, G/C -> A/T substitutions are categorized as S -> W, and A/T -> G/C as W -> S substitutions. Substitutions that do not alter the GC-content in the gene, S -> S or W -> W, were grouped together and are referred to as GC-conservative substitutions. To infer lineage-specific substitution rates for the different substitution categories, we applied a probabilistic mapping approach as implemented in the Bio++ library programs *BppML* and *MapNH* v2.3.2 (Dutheil and Boussau 2008; Romiguier et al. 2012). First, *BppML* was used to infer substitution model parameters. As estimated from the data, the best-fitting model was YN98 with F(3X4). These model parameters were then used with *MapNH* to infer *d_N_* and *d_S_* while correcting for nonstationarity, that is allowing GC-content to vary between lineages and taking ancestral nucleotide frequencies into account. Two models were applied per branch and gene; a branch model without accounting for substitution category and a model that infers specific substitution rates for each category. The number of substitutions was normalized by using the number of expected substitutions per category with the same substitution model, as implemented in the nullModelParams-option in *mapNH* (Guéguen and Duret 2018). *MapNH* does not handle missing data in the alignment resulting in a reduction in the number of genes with length >300 bp to 3,308 genes for these specific analyses. The differences in *d_N_*, *d_S_*, and ω estimates, between global substitution categories on the one hand and GC-conservative substitutions on the other, were tested with paired Wilcoxon signed rank test as implemented in R v.3.3.3 (R Core Team 2021).

### Gene Ontology Enrichment Analysis

To compare potential functional categories associated with specific gene sets, gene ontology (GO) enrichment analyses were performed. The analyses were applied to the aligned gene set and the 100 most conserved genes across Lepidoptera. Potential overrepresentation of specific functional categories in the classes biological process, molecular function, and cellular component were quantified using *PantherGO* (Mi et al. 2019). The Fisher's exact test implemented in the software evaluates over- or underrepresentation of ontology terms associated with a specific gene set as compared to a reference gene set included in the analysis. All annotated genes in *Drosophila melanogaster* in the Panther database were used as reference for the aligned gene set, and the aligned gene set was used as reference for the conserved gene set. The test statistic for each gene set was corrected for multiple testing with FDR correction (adjusted *P* value < 0.05), as implemented in the software.

### Characterization of Base Composition

To investigate if nucleotide composition was associated with evolutionary rates, codon position-specific proportions of GC were estimated in each species in each focal gene set (see above), and in the entire gene set of each lineage (CDS). Gene-wise GC-content was estimated for each position in each lineage and the differences between gene sets, and differences in GC-content between codon positions, were tested with pairwise Wilcoxon signed-rank tests. Differences in GC-content between codon positions within the gene sets were tested with a Kruskal–Wallis test, a nonparametric test for determining if multiple samples originate from the same distribution. Potential associations between gene-wise average GC-content and *d_N_*, *d_S_*, and ω were analyzed with Spearman's rank correlation test. The mean and standard deviation in lineage-specific GC-content in third codon positions were calculated from gene-wise nucleotide proportions and a potential association between mean and standard deviation was tested with Spearman's rank correlation test.

### Population Genetic Analysis of Mutation- and Fixation Bias in Leptidea Sinapis

We used previously published population resequencing data from 10 Swedish male *L. sinapis* individuals to investigate the S -> W/W -> S mutation bias and W -> S fixation bias at 0- and 4-fold degenerate sites. For more details on sequencing and variant calling, see Talla et al. (2019a). First, we polarized the alleles using parsimony by comparing the *L. sinapis* alleles with one individual from each of the closest relatives *Leptidea reali* and *Leptidea juvernica*. Then, we separated SNPs according to mutation category and computed derived allele frequency spectra for S, W, and GC-conservative mutation classes. Finally, we inferred the strength of the S -> W/W -> S mutation bias and the strength of fixation bias favoring GC (B = 4*N_e_*b, where b is analogous to the selection coefficient) using a population genetic model assuming gBGC-mutation-drift equilibrium (Glémin et al. 2015).

### Analysis of Codon Usage Frequencies

In order to characterize potential differences in codon frequencies between gene sets and lineages, we estimated the frequencies of all possible 61 amino acid encoding codons (stop codons ignored) using the *cusp* application as implemented in *EMBOSS* v.6.6.0 (Rice et al. 2000). The estimates of explicit codon frequencies were averaged per 1,000 codons for each of the lineages separately—a commonly applied procedure in codon frequency analysis (Clarke and Clark 2008; Athey et al. 2017). The relative synonymous codon usage (RSCU) was calculated by dividing the frequency of a specific codon with the expected frequency assuming that all synonymous codons would be equally frequently used for each amino acid (RSCU = 1) (Sharp and Li 1986). To assess if preferred codons were ending with G/C or A/T, overrepresented codons (RSCU >1) were categorized based on the nucleotide in the 3rd codon position in each lineage.

### Quantification of tRNA-Gene Copy Number

To explore the influence of tRNA abundance on codon usage bias in *L. sinapis*, we used the distribution of tRNA-gene copy number as proxy for tRNA abundance from a chromosome-level genome assembly from the Wellcome Sanger Institute Tree of Life initiative https://ftp.ncbi.nlm.nih.gov/genomes/all/GCF/905/404/315/GCF_905404315.1_ilLepSina1.1/GCF_905404315.1_ilLepSina1.1_genomic.fna.gz (Lohse et al. 2022). Only chromosome-level scaffolds were used for the analysis. The tRNA-gene models were detected with *tRNAscan-SE* version 2.0.9 for each chromosome with default settings (Chan et al. 2021). A custom script was used to remove truncated gene models and pseudogenes. We calculated the number of tRNA-gene copies for each specific anticodon, the number of tRNA-gene copies per aminoacid (isoacceptor tRNAs), and the relative abundance of isoacceptor tRNA (RAIT), or the relative use of specific anticodons per amino acid, which is equivalent to RSCU.

### Effective Number of Codons

To obtain a 1D estimate of codon usage per gene and lineage, we estimated the effective number of codons (ENC)—which theoretically ranges from 20 (extreme codon usage bias) to 61 (all codons used at equal frequencies)—using the *chips* application in *EMBOSS* v.6.6.0 (Rice et al. 2000). Codon usage bias can be caused by compositional constraint or selection on specific codons and to differentiate between these two processes we estimated the expected ENC based on composition alone (without selection). The expected ENC (ENC_exp_) was approximated based on base composition, using a modification of the formula:


$$\mathrm{EN}C_{\exp}=(a+GC_{3}+b)/(GC_{3}^{2}+(c\text{-}GC_{3}{)}^{2}),$$

where GC_3_ is the GC-content at third codon position (Wright 1990). We used optimized constants as suggested by simulations (a = 6, b = 34, c = 1.025) (dos Reis et al. 2004). In genes where synonymous codon usage is only affected by mutation, ENC_obs_ will be similar to the ENC_exp_ (Gun et al. 2018). The normalized difference, ENC_diff_ = (ENC_exp_–ENC_obs_)/ENC_exp_, was used as a proxy for the level of codon usage bias. Potential associations between ENC_obs_ and ENC_exp_, and the substitution rates, were estimated with nonparametric Spearman's rank correlation tests as implemented in R v.3.3.3 (https://www.R-project.org/).

### Linear Regression Analysis

To quantify the overall association and relative effects of base composition and codon usage on the evolutionary rate estimates in the different butterfly lineages, a multiple linear regression was performed for each lineage independently. The response variable (ω), was log-transformed to approach a normal distribution and GC_3_, ENC_obs_, and ENC_diff_ were included as explanatory variables. To accommodate for different scales, the explanatory variables were centered and scaled by subtracting with the mean and dividing by the standard deviation.

All statistical analyses described were performed in R v.3.3.3 (R Core Team 2021).

## Supplementary Material

evad150_Supplementary_Data

## Data Availability

The data used in this article are from public repositories. The genome and population data for *L. sinapis* are deposited on the European Nucleotide Archive (ENA) under accession number: PRJEB21838. The other genomes are available at LepBase http://ensembl.lepbase.org/. The scrips are available on GitHub (https://github.com/karinnasvall/GC_codon_usage_subst_GBE_2023).

## References

[evad150-B1] Altschul SF , Gish W, Miller W, Myers EW, Lipman DJ. 1990. Basic local alignment search tool. J Mol Biol. 215:403–410. 2231712 10.1016/S0022-2836(05)80360-2

[evad150-B2] Athey J , et al 2017. A new and updated resource for codon usage tables. BMC Bioinformatics. 18:391. 28865429 10.1186/s12859-017-1793-7 PMC5581930

[evad150-B3] Barton HJ , Zeng K. 2021. The effective population size modulates the strength of GC biased gene conversion in two passerines. bioRxiv (preprint). 2021.04.20.440602. doi: 10.1101/2021.04.20.440602, preprint: not peer reviewed.

[evad150-B4] Bates D , Mächler M, Bolker B, Walker S. 2015. Fitting linear mixed-effects models using lme4. J Stat Soft. 67(1):1–48.

[evad150-B5] Behura SK , Severson DW. 2011. Coadaptation of isoacceptor tRNA genes and codon usage bias for translation efficiency in *Aedes aegypti* and *Anopheles gambiae*. Insect Mol Biol. 20:177–187. 21040044 10.1111/j.1365-2583.2010.01055.x PMC3057532

[evad150-B6] Behura SK , Severson DW. 2012. Comparative analysis of codon usage bias and codon context patterns between dipteran and hymenopteran sequenced genomes. PLoS One. 7:e43111. 10.1371/journal.pone.0043111 PMC3422295 22912801

[evad150-B7] Bierne N , Eyre-Walker A. 2003. The problem of counting sites in the estimation of the synonymous and nonsynonymous substitution rates: implications for the correlation between the synonymous substitution rate and codon usage bias. Genetics 165:1587–1597. 14668405 10.1093/genetics/165.3.1587 PMC1462865

[evad150-B8] Boggs CL , Watt WB, Ehrlich PR. 2003. Butterflies: ecology and evolution taking flight. Chicago (IL): University of Chicago Press.

[evad150-B9] Bolívar P , et al 2018. Biased inference of selection due to GC-biased gene conversion and the rate of protein evolution in flycatchers when accounting for it. Mol Biol Evol. 35:2475–2486. 30085180 10.1093/molbev/msy149 PMC6188562

[evad150-B10] Bolívar P , Guéguen L, Duret L, Ellegren H, Mugal CF. 2019. GC-biased gene conversion conceals the prediction of the nearly neutral theory in avian genomes. Genome Biol. 20:5. 30616647 10.1186/s13059-018-1613-z PMC6322265

[evad150-B11] Boman J , Mugal CF, Backström N. 2021. The effects of GC-biased gene conversion on patterns of genetic diversity among and across butterfly genomes. Genome Biol Evol. 13:evab064. 10.1093/gbe/evab064 PMC8175052 33760095

[evad150-B12] Booker TR , Jackson BC, Keightley PD. 2017. Detecting positive selection in the genome. BMC Biol. 15:98. 29084517 10.1186/s12915-017-0434-y PMC5662103

[evad150-B13] Capella-Gutierrez S , Silla-Martinez JM, Gabaldon T. 2009. Trimal: a tool for automated alignment trimming in large-scale phylogenetic analyses. Bioinformatics 25:1972–1973. 19505945 10.1093/bioinformatics/btp348 PMC2712344

[evad150-B14] Challis RJ , Kumar S, Dasmahapatra KK, Jiggins CD, Blaxter M. 2016. Lepbase: the Lepidopteran genome database. 056994. doi: 10.1101/056994

[evad150-B15] Chan PP , Lin BY, Mak AJ, Lowe TM. 2021. tRNAscan-SE 2.0: improved detection and functional classification of transfer RNA genes. Nucleic Acids Res. 49:9077–9096. 34417604 10.1093/nar/gkab688 PMC8450103

[evad150-B16] Clarke TF , Clark PL. 2008. Rare codons cluster. PLoS One. 3:e3412. 18923675 10.1371/journal.pone.0003412 PMC2565806

[evad150-B17] Cong Q , Borek D, Otwinowski Z, Grishin NV. 2015a. Skipper genome sheds light on unique phenotypic traits and phylogeny. BMC Genomics. 16:639. 26311350 10.1186/s12864-015-1846-0 PMC4551732

[evad150-B18] Cong Q , Borek D, Otwinowski Z, Grishin NV. 2015b. Tiger swallowtail genome reveals mechanisms for speciation and caterpillar chemical defense. Cell Rep. 10:910–919. 25683714 10.1016/j.celrep.2015.01.026 PMC8935626

[evad150-B19] Cong Q , Shen J, Borek D, et al 2016. Complete genomes of hairstreak butterflies, their speciation and nucleo-mitochondrial incongruence. Sci Rep. 6:24863. 27120974 10.1038/srep24863 PMC4848470

[evad150-B20] Cong Q , Shen J, Warren AD, et al 2016. Speciation in cloudless sulphurs gleaned from complete genomes. Genome Biol Evol. 8:915–931. 26951782 10.1093/gbe/evw045 PMC4894063

[evad150-B21] Davey JW , et al 2016. Major improvements to the heliconius melpomene genome assembly used to confirm 10 chromosome fusion events in 6-million years of butterfly evolution . G3 (Bethesda). 6:695–708. 26772750 10.1534/g3.115.023655 PMC4777131

[evad150-B22] Dennis AB , et al 2020. Functional insights from the GC-poor genomes of two aphid parasitoids, *Aphidius ervi* and *Lysiphlebus fabarum*. BMC Genomics. 21:376. 32471448 10.1186/s12864-020-6764-0 PMC7257214

[evad150-B23] dos Reis M , Savva R, Wernisch L. 2004. Solving the riddle of codon usage preferences: a test for translational selection. Nucleic Acids Res. 32:5036–5044. 15448185 10.1093/nar/gkh834 PMC521650

[evad150-B24] Duret L . 2000. tRNA gene number and codon usage in the *C. elegans* genome are co-adapted for optimal translation of highly expressed genes. Trends Genet. 16:287–289. 10858656 10.1016/s0168-9525(00)02041-2

[evad150-B25] Duret L , Galtier N. 2009. Biased gene conversion and the evolution of mammalian genomic landscapes. Annu Rev Genom Hum Genet. 10:285–311. 10.1146/annurev-genom-082908-150001 19630562

[evad150-B26] Dutheil J , Boussau B. 2008. Non-homogeneous models of sequence evolution in the Bio++ suite of libraries and programs. BMC Evol Biol. 8:255. 18808672 10.1186/1471-2148-8-255 PMC2559849

[evad150-B27] Espeland M , et al 2018. A comprehensive and dated phylogenomic analysis of butterflies. Curr Biol. 28:770–778.e5. 29456146 10.1016/j.cub.2018.01.061

[evad150-B28] Figuet E , Ballenghien M, Romiguier J, Galtier N. 2015. Biased gene conversion and gc-content evolution in the coding sequences of reptiles and vertebrates. Genome Biol Evol. 7:240–250. 10.1093/gbe/evu277 PMC4316630 25527834

[evad150-B29] Frohlich DR , Wells MA. 1994. Codon usage patterns among genes for lepidopteran hemolymph proteins. J Mol Evol. 38:476–481. 8028026 10.1007/BF00178847

[evad150-B30] Galtier N . 2016. Adaptive protein evolution in animals and the effective population size hypothesis. PLoS Genet. 12:e1005774. 10.1371/journal.pgen.1005774 PMC4709115 26752180

[evad150-B31] Galtier N , et al 2018. Codon usage bias in animals: disentangling the effects of natural selection, effective population size, and GC-biased gene conversion. Mol Biol Evol. 35:1092–1103. 29390090 10.1093/molbev/msy015

[evad150-B32] Garel J-P . 1974. Functional adaptation of tRNA population. J Theor Biol. 43:211–225. 4591738 10.1016/s0022-5193(74)80054-8

[evad150-B33] Glémin S , et al 2015. Quantification of GC-biased gene conversion in the human genome. Genome Res. 25:1215–1228. 25995268 10.1101/gr.185488.114 PMC4510005

[evad150-B34] Guéguen L , Duret L. 2018. Unbiased estimate of synonymous and nonsynonymous substitution rates with nonstationary base composition. Mol Biol Evol. 35:734–742. 29220511 10.1093/molbev/msx308 PMC5850866

[evad150-B35] Gun L , Yumio R, Haixian P, Liang Z. 2018. Comprehensive analysis and comparison on the codon usage pattern of whole *Mycobacterium tuberculosis* coding genome from different area. BioMed Res Int. 2018:1–7. 10.1155/2018/3574976 PMC5964552 29854746

[evad150-B36] Higgs PG , Ran W. 2008. Coevolution of codon usage and tRNA genes leads to alternative stable states of biased codon usage. Mol Biol Evol. 25:2279–2291. 18687657 10.1093/molbev/msn173

[evad150-B37] Iijima T , et al 2018. Parallel evolution of Batesian mimicry supergene in two *Papilio* butterflies. *P. polytes* and *P. memnon*. Sci Adv. 4:eaao5416. 10.1126/sciadv.aao5416 PMC5906075 29675466

[evad150-B38] Ikemura T . 1982. Correlation between the abundance of yeast transfer RNAs and the occurrence of the respective codons in protein genes: differences in synonymous codon choice patterns of yeast and *Escherichia coli* with reference to the abundance of isoaccepting transfer RNAs. J Mol Biol. 158:573–597. 6750137 10.1016/0022-2836(82)90250-9

[evad150-B39] i Torres AP , et al 2023. The fine-scale recombination rate variation and associations with genomic features in a butterfly. Genome Res. 33:810–823. 37308293 10.1101/gr.277414.122 PMC10317125

[evad150-B40] Jørgensen FG , Schierup MH, Clark AG. 2007. Heterogeneity in regional GC content and differential usage of codons and amino acids in GC-poor and GC-rich regions of the genome of *Apis mellifera*. Mol Biol Evol. 24:611–619. 17150976 10.1093/molbev/msl190

[evad150-B41] Kanaya S , Yamada Y, Kudo Y, Ikemura T. 1999. Studies of codon usage and tRNA genes of 18 unicellular organisms and quantification of *Bacillus subtilis* tRNAs: gene expression level and species-specific diversity of codon usage based on multivariate analysis. Gene 238:143–155. 10570992 10.1016/s0378-1119(99)00225-5

[evad150-B42] Kawahara AY , et al 2019. Phylogenomics reveals the evolutionary timing and pattern of butterflies and moths. Proc Natl Acad Sci U S A. 116:22657–22663. 31636187 10.1073/pnas.1907847116 PMC6842621

[evad150-B43] Kawakami T , et al 2019. Substantial heritable variation in recombination rate on multiple scales in honeybees and bumblebees. Genetics 212:1101–1119. 31152071 10.1534/genetics.119.302008 PMC6707477

[evad150-B44] Kawamoto M , et al 2019. High-quality genome assembly of the silkworm, *Bombyx mori*. Insect Biochem Mol Biol. 107:53–62. 30802494 10.1016/j.ibmb.2019.02.002

[evad150-B45] Keightley PD , et al 2015. Estimation of the spontaneous mutation rate in *Heliconius melpomene*. Mol Biol Evol. 32:239–243. 25371432 10.1093/molbev/msu302 PMC4271535

[evad150-B46] Kimura M . 1977. Preponderance of synonymous changes as evidence for the neutral theory of molecular evolution. Nature 267:275–276. 865622 10.1038/267275a0

[evad150-B47] Knight RD , Freeland SJ, Landweber LF. 2001. A simple model based on mutation and selection explains trends in codon and amino-acid usage and GC composition within and across genomes. Genome Biol. 2:RESEARCH0010. 10.1186/gb-2001-2-4-research0010 PMC31479 11305938

[evad150-B48] Lamolle G , Fontenla S, Rijo G, Tort JF, Smircich P. 2019. Compositional analysis of flatworm genomes shows strong codon usage biases across all classes. Front Genet. 10:771. 31543897 10.3389/fgene.2019.00771 PMC6739440

[evad150-B49] Lanfear R , Thomas JA, Welch JJ, Brey T, Bromham L. 2007. Metabolic rate does not calibrate the molecular clock. Proc Natl Acad Sci U S A. 104:15388–15393. 17881572 10.1073/pnas.0703359104 PMC2000532

[evad150-B50] Li W , et al 2019. Genomes of skipper butterflies reveal extensive convergence of wing patterns. Proc Natl Acad Sci U S A. 116:6232–6237. 30877254 10.1073/pnas.1821304116 PMC6442542

[evad150-B51] Liu Y . 2020. A code within the genetic code: codon usage regulates co-translational protein folding. Cell Commun Signal. 18:145. 32907610 10.1186/s12964-020-00642-6 PMC7488015

[evad150-B52] Lobry JR , Gautier C. 1994. Hydrophobicity, expressivity and aromaticity are the major trends of amino-acid usage in 999 *Escherichia coli* chromosome-encoded genes. Nucleic Acids Res. 22:3174–3180. 8065933 10.1093/nar/22.15.3174 PMC310293

[evad150-B53] Lohse K , et al 2022. The genome sequence of the wood white butterfly *Leptidea sinapis* (Linnaeus, 1758). Wellcome Open Res. 7:254 37025368 10.12688/wellcomeopenres.18118.1 PMC10071140

[evad150-B54] Long H , et al 2018. Evolutionary determinants of genome-wide nucleotide composition. Nat Ecol Evol. 2:237–240. 29292397 10.1038/s41559-017-0425-y PMC6855595

[evad150-B55] Löytynoja A . 2014. Phylogeny-aware alignment with PRANK. Methods Mol Biol. 1079:155–170. 24170401 10.1007/978-1-62703-646-7_10

[evad150-B56] Lynch M . 2007. The origins of genome architecture. Sunderland (MA): Sinauer Associates.

[evad150-B57] Mackintosh A , et al 2019. The determinants of genetic diversity in butterflies. Nat Commun. 10:3466. 31371715 10.1038/s41467-019-11308-4 PMC6672018

[evad150-B58] Marais G . 2003. Biased gene conversion: implications for genome and sex evolution. Trends Genet. 19:330–338. 12801726 10.1016/S0168-9525(03)00116-1

[evad150-B59] Martin SH , et al 2016. Natural selection and genetic diversity in the butterfly *Heliconius melpomene*. Genetics 203:525–541. 27017626 10.1534/genetics.115.183285 PMC4858797

[evad150-B60] Maside X , Lee AW, Charlesworth B. 2004. Selection on codon usage in *Drosophila americana*. Curr Biol. 14:150–154. 14738738 10.1016/j.cub.2003.12.055

[evad150-B61] McVean GAT , Charlesworth B. 1999. A population genetic model for the evolution of synonymous codon usage: patterns and predictions. Genet Res. 74:145–158.

[evad150-B62] Mi H , Muruganujan A, Ebert D, Huang X, Thomas PD. 2019. PANTHER Version 14: more genomes, a new PANTHER GO-slim and improvements in enrichment analysis tools. Nucleic Acids Res. 47:D419–D426. 30407594 10.1093/nar/gky1038 PMC6323939

[evad150-B63] Moriyama EN , Powell JR. 1997. Codon usage bias and tRNA abundance in *Drosophila*. J Mol Evol. 45:514–523. 9342399 10.1007/pl00006256

[evad150-B64] Mugal CF , Weber CC, Ellegren H. 2015. GC-biased gene conversion links the recombination landscape and demography to genomic base composition: GC-biased gene conversion drives genomic base composition across a wide range of species. BioEssays 37:1317–1326. 26445215 10.1002/bies.201500058

[evad150-B65] Muyle A , Serres-Giardi L, Ressayre A, Escobar J, Glémin S. 2011. GC-biased gene conversion and selection affect GC content in the *Oryza* genus (rice). Mol Biol Evol. 28:2695–2706. 21504892 10.1093/molbev/msr104

[evad150-B66] Nabholz B , Glemin S, Galtier N. 2007. Strong variations of mitochondrial mutation rate across mammals–the longevity hypothesis. Mol Biol Evol. 25:120–130. 17998254 10.1093/molbev/msm248

[evad150-B67] Nagylaki T . 1983. Evolution of a finite population under gene conversion. Proc Natl Acad Sci U S A. 80:6278–6281. 6578508 10.1073/pnas.80.20.6278 PMC394279

[evad150-B68] Nielsen R . 2005. Molecular signatures of natural selection. Annu Rev Genet. 39:197–218. 16285858 10.1146/annurev.genet.39.073003.112420

[evad150-B69] Novoa EM , Jungreis I, Jaillon O, Kellis M. 2019. Elucidation of codon usage signatures across the domains of life. Mol Biol Evol. 36:2328–2339. 31220870 10.1093/molbev/msz124 PMC6759073

[evad150-B70] Ohta T . 1992. The nearly neutral theory of molecular evolution. Annu Rev Ecol Syst. 23:263–286.

[evad150-B71] Palidwor GA , Perkins TJ, Xia X. 2010. A general model of codon bias due to GC mutational bias. PLoS One. 5:e13431. 10.1371/journal.pone.0013431 PMC2965080 21048949

[evad150-B72] Percudani R , Pavesi A, Ottonello S. 1997. Transfer RNA gene redundancy and translational selection in *Saccharomyces cerevisiae*. J Mol Biol. 268:322–330. 9159473 10.1006/jmbi.1997.0942

[evad150-B73] Petrov DA , Hartl DL. 1999. Patterns of nucleotide substitution in *Drosophila* and mammalian genomes. Proc Natl Acad Sci U S A. 96:1475–1479. 9990048 10.1073/pnas.96.4.1475 PMC15487

[evad150-B74] Presnyak V , et al 2015. Codon optimality is a major determinant of mRNA stability. Cell 160:1111–1124. 25768907 10.1016/j.cell.2015.02.029 PMC4359748

[evad150-B75] Rao Y , et al 2011. Mutation bias is the driving force of codon usage in the *Gallus gallus* genome. DNA Res. 18:499–512. 22039174 10.1093/dnares/dsr035 PMC3223081

[evad150-B76] R Core Team . 2021. R: A language and environment for statistical computing. R Foundation for Statistical Computing, Vienna, Austria. Available from: http://www.r-project.org/index.html (Accessed March 23, 2022).

[evad150-B77] Rice P , Longden I, Bleasby A. 2000. EMBOSS: the European Molecular Biology Open Software Suite. Trends Genet. 16:276–277. 10827456 10.1016/s0168-9525(00)02024-2

[evad150-B78] Romiguier J , et al 2012. Fast and robust characterization of time-heterogeneous sequence evolutionary processes using substitution mapping. PLoS One. 7:e33852. 10.1371/journal.pone.0033852 PMC3313935 22479459

[evad150-B79] Sharma J , Chakraborty S, Uddin A. 2012. Comparative analysis of codon usage bias between two lepidopteran insect species: *Bombyx mandarina* and *Ostrinia furnacalis*. Int J Sci Res. 3:47–50.

[evad150-B80] Sharp PM . 2001. Codon usage bias. In: Brenner S, Miller JH, editors. Encyclopedia of genetics. New York (USA): Academic Press. p. 402–406.

[evad150-B81] Sharp PM , Li WH. 1986. Codon usage in regulatory genes in Escherichia coli does not reflect selection for ‘rare’ codons. Nucleic Acids Res. 14:7737–7749. 3534792 10.1093/nar/14.19.7737 PMC311793

[evad150-B82] Sharp PM , Li WH. 1989. On the rate of DNA sequence evolution in Drosophila. J Mol Evol. 28:398–402. 2501501 10.1007/BF02603075

[evad150-B83] Shields O . 1989. World numbers of butterflies. J Lepid Soc. 43:178–183.

[evad150-B84] Shipilina D , et al 2022. Linkage mapping and genome annotation give novel insights into gene family expansions and regional recombination rate variation in the painted lady (*Vanessa cardui*) butterfly. Genomics 114:110481. 10.1016/j.ygeno.2022.110481 36115505

[evad150-B85] Stamatakis A . 2014. RAxML version 8: a tool for phylogenetic analysis and post-analysis of large phylogenies. Bioinformatics 30:1312–1313. 24451623 10.1093/bioinformatics/btu033 PMC3998144

[evad150-B86] Stein LD , et al 2003. The genome sequence of *Caenorhabditis briggsae* : a platform for comparative genomics. PLoS Biol. 1:e45. 14624247 10.1371/journal.pbio.0000045 PMC261899

[evad150-B87] Stinchcombe JR , Hoekstra HE. 2008. Combining population genomics and quantitative genetics: finding the genes underlying ecologically important traits. Heredity (Edinb). 100:158–170. 17314923 10.1038/sj.hdy.6800937

[evad150-B88] Talla V , et al 2017. Rapid increase in genome size as a consequence of transposable element hyperactivity in wood-white (*Leptidea*) butterflies. Genome Biol Evol. 9:2491–2505. 28981642 10.1093/gbe/evx163 PMC5737376

[evad150-B89] Talla V , et al 2019. Dissecting the effects of selection and mutation on genetic diversity in three wood white (*Leptidea*) butterfly species. Genome Biol Evol. 11:2875–2886. 31580421 10.1093/gbe/evz212 PMC6795238

[evad150-B90] Thomas JA , Welch JJ, Lanfear R, Bromham L. 2010. A generation time effect on the rate of molecular evolution in invertebrates. Mol Biol Evol. 27:1173–1180. 20083649 10.1093/molbev/msq009

[evad150-B91] Thomas JA , Welch JJ, Woolfit M, Bromham L. 2006. There is no universal molecular clock for invertebrates, but rate variation does not scale with body size. Proc Natl Acad Sci U S A. 103:7366–7371. 16651532 10.1073/pnas.0510251103 PMC1464347

[evad150-B92] Tolman T , Lewington R. 2009. Collins butterfly guide. New ed. London: Collins.

[evad150-B93] Varenne S , Buc J, Lloubes R, Lazdunski C. 1984. Translation is a non-uniform process: effect of tRNA availability on the rate of elongation of nascent polypeptide chains. J Mol Biol. 180:549–576. 6084718 10.1016/0022-2836(84)90027-5

[evad150-B94] Vicario S , Moriyama EN, Powell JR. 2007. Codon usage in twelve species of *Drosophila*. BMC Evol Biol. 7:226. 18005411 10.1186/1471-2148-7-226 PMC2213667

[evad150-B95] Wright F . 1990. The ‘effective number of codons’ used in a gene. Gene 87:23–29. 2110097 10.1016/0378-1119(90)90491-9

[evad150-B96] Yang Z . 2007. PAML 4: phylogenetic analysis by maximum likelihood. Mol Biol Evol. 24:1586–1591. 17483113 10.1093/molbev/msm088

[evad150-B97] Zhan S , Merlin C, Boore JL, Reppert SM. 2011. The monarch butterfly genome yields insights into long-distance migration. Cell 147:1171–1185. 22118469 10.1016/j.cell.2011.09.052 PMC3225893

[evad150-B98] Zhao F , Yu C, Liu Y. 2017. Codon usage regulates protein structure and function by affecting translation elongation speed in *Drosophila* cells. Nucleic Acids Res. 45:8484–8492. 28582582 10.1093/nar/gkx501 PMC5737824

[evad150-B99] Zhou Z , et al 2016. Codon usage is an important determinant of gene expression levels largely through its effects on transcription. Proc Natl Acad Sci U S A. 113:E6117–E6125. 27671647 10.1073/pnas.1606724113 PMC5068308

