# Supplementary material for: Base Composition, Codon Usage, and Patterns of Gene Sequence Evolution in Butterflies
Source: Genome Biol Evol. 2023 Aug 11;15(8):evad150. doi: 10.1093/gbe/evad150 (PMC10462419; doi:10.1093/gbe/evad150)
Supplement: evad150_Supplementary_Data [file evad150_supplementary_data.docx]

**Supplementary information**

Table of content

Supplementary figures …………1

Supplementary tables …………..9

Supplementary analysis ……….37

# Supplementary figures

**Supplementary Figure 1.** The density distribution of gene specific ω estimates for the 4,150 1:1 orthologs across the eight Lepidoptera lineages (the ‘aligned gene set’). The x-axis shows ω and the y-axis the relative density for each ω bin (number of bins = 512). The curve has been smoothed.

**Supplementary Figure 2.** a) Generalised mixed linear model estimates (dots) and confidence intervals (whiskers) with d_N_/d_S_ as dependant variable, from PaML (light brown) and from mapNH (brown), using species as fixed variable and gene ID as random variable. b) Distributions of ω (y-axis, log_10_-transformed) as inferred in *codeml* (PaML) in the aligned gene set in the eight Lepidoptera lineages. Boxes represent first and third quartiles, the horizontal lines within boxes show median values, whiskers represent upper and lower values (1.5 * interquartile range) and the solid dots indicate outliers.

**a**

**Supplementary Figure 3.** a) Lineage specific distribution of gene-wise GC-content in each codon position. The dashed vertical line represents 50% GC. Lineage specific associations between GC-content in third codon position and a) ω, b) synonymous (*d_S_*), and, c) non-synonymous (*d_N_*) substitution rates for all, GC-conservative (GC-cons), GC-decreasing (S -> W) and GC-increasing (W -> S) substitutions. The lines represent local linear regression models (LOESS) applied to each respective analysis.

**Supplementary Figure 4.** Violin plot of the lineage specific distributions of relative synonymous codon usage (RSCU) per nucleotide at third codon positions. The horizontal dashed line represents neutral RSCU, i.e. that all codons are used at the same frequency.

**Supplementary Figure 5.** Lineage specific a) observed codon usage (ENC_obs_, y-axis) and b) proportional difference between expected and observed ENC (ENC_diff_) per gene as a function of GC-content in third codon positions. Lines represent linear regressions. Spearman’s rho and p-values are included. c) Lineage specific distribution of ENC_obs_, and d) ENC_diff_ per dataset; the 100 most conserved (lowest ω) and the rest of the aligned data set (neutral). Boxes represent first and third quartiles, the horizontal lines within boxes show median values, whiskers represent upper and lower values (1.5 * interquartile range) and the solid dots indicate outliers. The p-values reflect Wilcoxon’s rank sum tests from each respective comparison.

# Supplementary tables

**Supplementary Table 1.** List of taxa included in the analysis, the corresponding genome assembly size and number of annotated genes (# Genes) for each respective species, and the proportion (%) of 1:1 orthologs (Orthologs) and aligned genes (Aligned) used for subsequent analysis. Date aq. = date when the data was downloaded.

| Species | Date aq. | Size (Mb) | # Genes | Orthologs | Aligned | GC-content |
| --- | --- | --- | --- | --- | --- | --- |
| Bombyx_mori_ASM15162v1 | 20180216 | 481.8 | 19618 | 34.0 | 19.5 | 37.7 |
| Papilio_machaon_Pap_ma_1.0 | 20180221 | 278.4 | 15497 | 43.1 | 24.7 | 33.8 |
| Lerema_accius_v1.1 | 20180221 | 298.2 | 17411 | 38.3 | 21.9 | 31.6 |
| Leptidea_sinapis_V1.1 | In house | 643.0 | 15598 | 42.8 | 24.5 | 34.4 |
| Phoebis_sennae_v1.1 | 20180221 | 345.4 | 16492 | 40.5 | 23.2 | 33.0 |
| Calycopis_cecrops_v1.1 | 20180409 | 728.8 | 16456 | 40.6 | 23.2 | 37.1 |
| Danaus_plexippus_v3 | 20180221 | 248.6 | 15130 | 44.1 | 25.3 | 31.6 |
| Heliconius_melpomene_melpomene_Hmel2 | 20180221 | 275.2 | 21661 | 30.8 | 17.6 | 32.8 |

**Supplementary Table 2.** GC-content in percent overall and at each codon position for all lineages, for each dataset. The test statistic (Test W) and p-value from Wilcoxon rank sum test of the difference in average GC-content between datasets (aligned gene set vs CDS and conserved gene set).

| Pos | Dataset | All | +/-Sd | *B_mor* | *C_cec* | *D_ple* | *H_mel* | *L_acc* | *L_sin* | *P_mac* | *P_sen* | Test W | p-value |
| --- | --- | --- | --- | --- | --- | --- | --- | --- | --- | --- | --- | --- | --- |
| All | CDS | 46,60 | 1,51 | 48,27 | 48,87 | 46,45 | 44,34 | 46,51 | 44,98 | 47,06 | 46,28 | 44 | 2,35E-01 |
|  | Align | 45,70 | 1,57 | 46,03 | 48,06 | 45,43 | 42,87 | 46,98 | 44,42 | 45,88 | 46,05 | NA | NA |
|  | Cons | 45,22 | 1,18 | 45,20 | 45,15 | 45,50 | 43,36 | 47,26 | 43,88 | 45,56 | 45,69 | 195226 | 3,11E-01 |
| 1st | CDS | 51,35 | 0,70 | 52,03 | 52,25 | 51,25 | 50,65 | 50,92 | 50,46 | 52,13 | 51,08 | 38 | 5,74E-01 |
|  | Align | 51,16 | 0,65 | 51,21 | 51,90 | 50,82 | 50,04 | 51,85 | 50,57 | 51,65 | 51,18 | NA | NA |
|  | Cons | 52,49 | 0,39 | 52,50 | 52,60 | 52,61 | 51,93 | 53,20 | 52,06 | 52,54 | 52,45 | 0 | 1,55E-04 |
| 2nd | CDS | 40,29 | 0,51 | 41,29 | 40,09 | 40,27 | 40,38 | 40,07 | 39,82 | 40,68 | 39,69 | 64 | 9,31E-04 |
|  | Align | 39,12 | 0,15 | 39,20 | 39,20 | 39,08 | 38,89 | 39,24 | 38,94 | 39,30 | 39,06 | NA | NA |
|  | Cons | 37,50 | 0,07 | 37,45 | 37,49 | 37,42 | 37,55 | 37,39 | 37,42 | 37,45 | 37,61 | 64 | 1,55E-04 |
| 3rd | CDS | 48,15 | 3,76 | 51,48 | 54,27 | 47,84 | 41,98 | 48,54 | 44,66 | 48,36 | 48,07 | 41 | 3,82E-01 |
|  | Align | 46,81 | 3,98 | 47,68 | 53,07 | 46,38 | 39,67 | 49,86 | 43,74 | 46,68 | 47,92 | NA | NA |
|  | Cons | 45,65 | 3,20 | 45,65 | 45,37 | 46,47 | 40,62 | 51,18 | 42,15 | 46,70 | 47,00 | 40 | 4,42E-01 |

**Supplementary Table 3.** List of gene ontology terms significantly over or underrepresented in the aligned gene set (n = 4,150 genes, only 1,863 with functional annotation), only the 50 terms with highest enrichment are included. Comparison against the *Drosophila melanogaster* reference list (#ref set) in Panther GO-slim (n=13,811). The table contains numbers of observed (# obs) and expected (# exp) occurrences of the GO-term in the aligned gene set. Over- or underrepresentation is expressed as positive or negative fold change (Fold). pFDR is the p-value after FDR correction.

| **PANTHER GO-Slim Biological Process** | **#ref set** | **#obs** | **#exp** | **fold** | **pFDR** |
| --- | --- | --- | --- | --- | --- |
| regulation of stress-activated MAPK cascade (GO:0032872) | 8 | 6 | 1.08 | 5.56 | 2.65E-02 |
| regulation of stress-activated protein kinase signaling cascade (GO:0070302) | 8 | 6 | 1.08 | 5.56 | 2.64E-02 |
| regulation of mitotic sister chromatid separation (GO:0010965) | 12 | 8 | 1.62 | 4.94 | 1.19E-02 |
| metaphase/anaphase transition of mitotic cell cycle (GO:0007091) | 12 | 8 | 1.62 | 4.94 | 1.19E-02 |
| stress-activated MAPK cascade (GO:0051403) | 9 | 6 | 1.21 | 4.94 | 3.55E-02 |
| regulation of mitotic nuclear division (GO:0007088) | 15 | 10 | 2.02 | 4.94 | 3.66E-03 |
| regulation of nuclear division (GO:0051783) | 17 | 11 | 2.29 | 4.8 | 2.47E-03 |
| regulation of mitotic metaphase/anaphase transition (GO:0030071) | 11 | 7 | 1.48 | 4.72 | 2.45E-02 |
| ribosomal large subunit assembly (GO:0000027) | 19 | 12 | 2.56 | 4.68 | 1.60E-03 |
| regulation of chromosome separation (GO:1905818) | 13 | 8 | 1.75 | 4.56 | 1.61E-02 |
| regulation of mitotic sister chromatid segregation (GO:0033047) | 13 | 8 | 1.75 | 4.56 | 1.60E-02 |
| mitotic sister chromatid separation (GO:0051306) | 13 | 8 | 1.75 | 4.56 | 1.60E-02 |
| cell cycle DNA replication (GO:0044786) | 13 | 8 | 1.75 | 4.56 | 1.59E-02 |
| metaphase/anaphase transition of cell cycle (GO:0044784) | 13 | 8 | 1.75 | 4.56 | 1.58E-02 |
| nuclear DNA replication (GO:0033260) | 13 | 8 | 1.75 | 4.56 | 1.58E-02 |
| regulation of sister chromatid segregation (GO:0033045) | 15 | 9 | 2.02 | 4.45 | 1.06E-02 |
| regulation of chromosome segregation (GO:0051983) | 15 | 9 | 2.02 | 4.45 | 1.05E-02 |
| histone H3 acetylation (GO:0043966) | 10 | 6 | 1.35 | 4.45 | 4.66E-02 |
| regulation of metaphase/anaphase transition of cell cycle (GO:1902099) | 12 | 7 | 1.62 | 4.32 | 3.06E-02 |
| stress-activated protein kinase signaling cascade (GO:0031098) | 14 | 8 | 1.89 | 4.24 | 2.10E-02 |
| endosome transport via multivesicular body sorting pathway (GO:0032509) | 16 | 9 | 2.16 | 4.17 | 1.39E-02 |
| DNA replication initiation (GO:0006270) | 18 | 10 | 2.43 | 4.12 | 9.00E-03 |
| cellular response to BMP stimulus (GO:0071773) | 13 | 7 | 1.75 | 3.99 | 4.02E-02 |
| response to BMP (GO:0071772) | 13 | 7 | 1.75 | 3.99 | 4.01E-02 |
| BMP signaling pathway (GO:0030509) | 13 | 7 | 1.75 | 3.99 | 4.00E-02 |
| regulation of cellular response to stress (GO:0080135) | 20 | 10 | 2.7 | 3.71 | 1.50E-02 |
| regulation of response to stress (GO:0080134) | 20 | 10 | 2.7 | 3.71 | 1.49E-02 |
| intra-Golgi vesicle-mediated transport (GO:0006891) | 21 | 10 | 2.83 | 3.53 | 1.84E-02 |
| multivesicular body sorting pathway (GO:0071985) | 19 | 9 | 2.56 | 3.51 | 2.76E-02 |
| retrograde transport, endosome to Golgi (GO:0042147) | 19 | 9 | 2.56 | 3.51 | 2.75E-02 |
| transmembrane receptor protein serine/threonine kinase signaling pathway (GO:0007178) | 19 | 9 | 2.56 | 3.51 | 2.74E-02 |
| late endosome to vacuole transport (GO:0045324) | 17 | 8 | 2.29 | 3.49 | 4.07E-02 |
| regulation of mitotic cell cycle phase transition (GO:1901990) | 30 | 14 | 4.05 | 3.46 | 4.17E-03 |
| regulation of cell cycle process (GO:0010564) | 41 | 19 | 5.53 | 3.44 | 5.58E-04 |
| endosomal transport (GO:0016197) | 52 | 24 | 7.01 | 3.42 | 7.47E-05 |
| mitotic cell cycle phase transition (GO:0044772) | 39 | 18 | 5.26 | 3.42 | 8.95E-04 |
| regulation of cell cycle phase transition (GO:1901987) | 31 | 14 | 4.18 | 3.35 | 5.24E-03 |
| regulation of MAPK cascade (GO:0043408) | 20 | 9 | 2.7 | 3.34 | 3.34E-02 |
| cell cycle phase transition (GO:0044770) | 40 | 18 | 5.4 | 3.34 | 1.14E-03 |
| mitotic sister chromatid segregation (GO:0000070) | 29 | 13 | 3.91 | 3.32 | 8.16E-03 |
| peptide biosynthetic process (GO:0043043) | 183 | 82 | 24.69 | 3.32 | 4.63E-15 |
| response to growth factor (GO:0070848) | 18 | 8 | 2.43 | 3.29 | 4.98E-02 |
| cellular response to growth factor stimulus (GO:0071363) | 18 | 8 | 2.43 | 3.29 | 4.97E-02 |
| translational elongation (GO:0006414) | 181 | 80 | 24.42 | 3.28 | 1.67E-14 |
| translation (GO:0006412) | 181 | 80 | 24.42 | 3.28 | 1.62E-14 |
| cell surface receptor signaling pathway involved in cell-cell signaling (GO:1905114) | 32 | 14 | 4.32 | 3.24 | 6.54E-03 |
| MAPK cascade (GO:0000165) | 23 | 10 | 3.1 | 3.22 | 2.75E-02 |
| negative regulation of cell cycle process (GO:0010948) | 21 | 9 | 2.83 | 3.18 | 4.04E-02 |
| translational initiation (GO:0006413) | 28 | 12 | 3.78 | 3.18 | 1.50E-02 |
| cytosolic transport (GO:0016482) | 21 | 9 | 2.83 | 3.18 | 4.02E-02 |
|  |  |  |  |  |  |
|  |  |  |  |  |  |
|  |  |  |  |  |  |
| **PANTHER GO-Slim Cellular Component** | **#ref set** | **#obs** | **#exp** | **fold** | **pFDR** |
| nuclear origin of replication recognition complex (GO:0005664) | 6 | 5 | 0.81 | 6.18 | 3.64E-02 |
| recycling endosome (GO:0055037) | 8 | 6 | 1.08 | 5.56 | 2.55E-02 |
| transcription factor TFIIH core complex (GO:0000439) | 8 | 6 | 1.08 | 5.56 | 2.52E-02 |
| transcription factor TFIIH holo complex (GO:0005675) | 8 | 6 | 1.08 | 5.56 | 2.48E-02 |
| proteasome regulatory particle, base subcomplex (GO:0008540) | 10 | 7 | 1.35 | 5.19 | 1.62E-02 |
| mitochondrial small ribosomal subunit (GO:0005763) | 12 | 8 | 1.62 | 4.94 | 1.10E-02 |
| eukaryotic translation initiation factor 3 complex (GO:0005852) | 15 | 9 | 2.02 | 4.45 | 9.84E-03 |
| cytosolic large ribosomal subunit (GO:0022625) | 46 | 27 | 6.21 | 4.35 | 4.24E-07 |
| large ribosomal subunit (GO:0015934) | 68 | 36 | 9.17 | 3.92 | 1.85E-08 |
| cytosolic ribosome (GO:0022626) | 84 | 44 | 11.33 | 3.88 | 4.62E-10 |
| ribosomal subunit (GO:0044391) | 120 | 62 | 16.19 | 3.83 | 9.42E-14 |
| small ribosomal subunit (GO:0015935) | 52 | 26 | 7.01 | 3.71 | 7.15E-06 |
| nuclear ubiquitin ligase complex (GO:0000152) | 14 | 7 | 1.89 | 3.71 | 4.67E-02 |
| ribosome (GO:0005840) | 134 | 67 | 18.08 | 3.71 | 2.66E-14 |
| organellar ribosome (GO:0000313) | 33 | 16 | 4.45 | 3.59 | 1.07E-03 |
| mitochondrial ribosome (GO:0005761) | 33 | 16 | 4.45 | 3.59 | 1.05E-03 |
| RNA polymerase II, holoenzyme (GO:0016591) | 46 | 22 | 6.21 | 3.55 | 8.91E-05 |
| origin recognition complex (GO:0000808) | 23 | 11 | 3.1 | 3.55 | 1.12E-02 |
| proteasome accessory complex (GO:0022624) | 19 | 9 | 2.56 | 3.51 | 2.60E-02 |
| proteasome regulatory particle (GO:0005838) | 19 | 9 | 2.56 | 3.51 | 2.57E-02 |
| mitochondrial matrix (GO:0005759) | 46 | 20 | 6.21 | 3.22 | 5.51E-04 |
| cytosolic small ribosomal subunit (GO:0022627) | 37 | 16 | 4.99 | 3.21 | 2.71E-03 |
| serine/threonine protein kinase complex (GO:1902554) | 26 | 11 | 3.51 | 3.14 | 2.14E-02 |
| Golgi-associated vesicle membrane (GO:0030660) | 22 | 9 | 2.97 | 3.03 | 4.64E-02 |
| protein kinase complex (GO:1902911) | 27 | 11 | 3.64 | 3.02 | 2.51E-02 |
| proteasome complex (GO:0000502) | 42 | 17 | 5.67 | 3.00 | 3.22E-03 |
| MCM core complex (GO:0097373) | 25 | 10 | 3.37 | 2.97 | 3.74E-02 |
| MCM complex (GO:0042555) | 25 | 10 | 3.37 | 2.97 | 3.69E-02 |
| DNA-directed RNA polymerase complex (GO:0000428) | 65 | 26 | 8.77 | 2.97 | 1.69E-04 |
| RNA polymerase complex (GO:0030880) | 66 | 26 | 8.9 | 2.92 | 2.94E-04 |
| nuclear DNA-directed RNA polymerase complex (GO:0055029) | 64 | 25 | 8.63 | 2.90 | 4.39E-04 |
| peptidase complex (GO:1905368) | 60 | 23 | 8.09 | 2.84 | 9.89E-04 |
| vesicle tethering complex (GO:0099023) | 37 | 14 | 4.99 | 2.81 | 1.47E-02 |
| endopeptidase complex (GO:1905369) | 46 | 17 | 6.21 | 2.74 | 9.89E-03 |
| RNA polymerase II transcription regulator complex (GO:0090575) | 84 | 30 | 11.33 | 2.65 | 3.03E-04 |
| centrosome (GO:0005813) | 48 | 17 | 6.47 | 2.63 | 1.17E-02 |
| Golgi-associated vesicle (GO:0005798) | 40 | 14 | 5.4 | 2.59 | 2.84E-02 |
| endosome (GO:0005768) | 93 | 32 | 12.55 | 2.55 | 2.40E-04 |
| vesicle membrane (GO:0012506) | 38 | 13 | 5.13 | 2.54 | 4.20E-02 |
| centriole (GO:0005814) | 53 | 18 | 7.15 | 2.52 | 1.15E-02 |
| cullin-RING ubiquitin ligase complex (GO:0031461) | 54 | 18 | 7.28 | 2.47 | 1.29E-02 |
| transferase complex, transferring phosphorus-containing groups (GO:0061695) | 110 | 36 | 14.84 | 2.43 | 1.89E-04 |
| ribonucleoprotein complex (GO:1990904) | 336 | 106 | 45.32 | 2.34 | 2.29E-11 |
| transferase complex (GO:1990234) | 282 | 88 | 38.04 | 2.31 | 1.96E-09 |
| cytoplasmic vesicle (GO:0031410) | 183 | 57 | 24.69 | 2.31 | 4.06E-06 |
| intracellular vesicle (GO:0097708) | 184 | 57 | 24.82 | 2.30 | 4.22E-06 |
| ubiquitin ligase complex (GO:0000151) | 72 | 22 | 9.71 | 2.27 | 1.45E-02 |
| vesicle (GO:0031982) | 197 | 60 | 26.57 | 2.26 | 3.74E-06 |
| cytosol (GO:0005829) | 434 | 126 | 58.54 | 2.15 | 2.34E-11 |
| actin cytoskeleton (GO:0015629) | 66 | 19 | 8.9 | 2.13 | 3.68E-02 |
|  |  |  |  |  |  |
|  |  |  |  |  |  |
|  |  |  |  |  |  |
| **PANTHER GO-Slim Molecular Function** | **#ref set** | **#obs** | **#exp** | **fold** | **pFDR** |
| 3'-5' DNA helicase activity (GO:0043138) | 12 | 8 | 1.62 | 4.94 | 2.24E-02 |
| structural constituent of ribosome (GO:0003735) | 109 | 65 | 14.7 | 4.42 | 3.85E-16 |
| DNA replication origin binding (GO:0003688) | 16 | 9 | 2.16 | 4.17 | 2.63E-02 |
| rRNA binding (GO:0019843) | 26 | 13 | 3.51 | 3.71 | 8.85E-03 |
| DNA helicase activity (GO:0003678) | 20 | 10 | 2.7 | 3.71 | 2.70E-02 |
| structural molecule activity (GO:0005198) | 145 | 72 | 19.56 | 3.68 | 8.91E-15 |
| single-stranded DNA binding (GO:0003697) | 25 | 11 | 3.37 | 3.26 | 3.18E-02 |
| G protein-coupled receptor binding (GO:0001664) | 23 | 10 | 3.1 | 3.22 | 4.40E-02 |
| guanyl-nucleotide exchange factor activity (GO:0005085) | 30 | 12 | 4.05 | 2.97 | 3.65E-02 |
| exonuclease activity (GO:0004527) | 31 | 12 | 4.18 | 2.87 | 4.02E-02 |
| translation initiation factor activity (GO:0003743) | 32 | 12 | 4.32 | 2.78 | 4.54E-02 |
| SNARE binding (GO:0000149) | 39 | 14 | 5.26 | 2.66 | 4.48E-02 |
| translation regulator activity (GO:0045182) | 55 | 19 | 7.42 | 2.56 | 1.70E-02 |
| ligase activity (GO:0016874) | 107 | 34 | 14.43 | 2.36 | 1.97E-03 |
| catalytic activity, acting on DNA (GO:0140097) | 68 | 21 | 9.17 | 2.29 | 2.58E-02 |
| RNA binding (GO:0003723) | 398 | 113 | 53.69 | 2.10 | 1.94E-09 |
| nuclease activity (GO:0004518) | 86 | 24 | 11.6 | 2.07 | 3.72E-02 |
| GTPase activity (GO:0003924) | 115 | 32 | 15.51 | 2.06 | 1.45E-02 |
| signaling receptor binding (GO:0005102) | 91 | 25 | 12.28 | 2.04 | 4.10E-02 |
| catalytic activity, acting on RNA (GO:0140098) | 170 | 45 | 22.93 | 1.96 | 4.43E-03 |
| protein-containing complex binding (GO:0044877) | 144 | 38 | 19.42 | 1.96 | 1.00E-02 |
| mRNA binding (GO:0003729) | 95 | 25 | 12.81 | 1.95 | 4.57E-02 |
| nucleic acid binding (GO:0003676) | 1025 | 242 | 138.3 | 1.75 | 5.63E-13 |
| heterocyclic compound binding (GO:1901363) | 1182 | 274 | 159.4 | 1.72 | 6.93E-14 |
| organic cyclic compound binding (GO:0097159) | 1196 | 276 | 161.3 | 1.71 | 6.09E-14 |
| phosphotransferase activity, alcohol group as acceptor (GO:0016773) | 258 | 59 | 34.8 | 1.70 | 9.21E-03 |
| protein binding (GO:0005515) | 901 | 203 | 121.5 | 1.67 | 4.05E-09 |
| protein kinase activity (GO:0004672) | 206 | 46 | 27.79 | 1.66 | 3.81E-02 |
| kinase activity (GO:0016301) | 290 | 63 | 39.12 | 1.61 | 1.64E-02 |
| transferase activity, transferring phosphorus-containing groups (GO:0016772) | 359 | 77 | 48.43 | 1.59 | 8.92E-03 |
| binding (GO:0005488) | 2402 | 515 | 324 | 1.59 | 3.12E-22 |
| double-stranded DNA binding (GO:0003690) | 534 | 112 | 72.03 | 1.55 | 1.06E-03 |
| DNA binding (GO:0003677) | 623 | 130 | 84.04 | 1.55 | 3.10E-04 |
| sequence-specific double-stranded DNA binding (GO:1990837) | 513 | 106 | 69.2 | 1.53 | 2.86E-03 |
| sequence-specific DNA binding (GO:0043565) | 528 | 109 | 71.22 | 1.53 | 2.24E-03 |
| molecular function regulator (GO:0098772) | 831 | 171 | 112.1 | 1.53 | 3.00E-05 |
| RNA polymerase II cis-regulatory region sequence-specific DNA binding (GO:0000978) | 347 | 71 | 46.81 | 1.52 | 3.13E-02 |
| cis-regulatory region sequence-specific DNA binding (GO:0000987) | 350 | 71 | 47.21 | 1.50 | 3.20E-02 |
| transcription regulator activity (GO:0140110) | 587 | 119 | 79.18 | 1.50 | 1.97E-03 |
| nucleoside-triphosphatase activity (GO:0017111) | 337 | 68 | 45.46 | 1.50 | 3.89E-02 |
| transferase activity (GO:0016740) | 888 | 175 | 119.8 | 1.46 | 1.90E-04 |
| transcription regulatory region sequence-specific DNA binding (GO:0000976) | 494 | 97 | 66.64 | 1.46 | 1.66E-02 |
| regulatory region nucleic acid binding (GO:0001067) | 494 | 97 | 66.64 | 1.46 | 1.61E-02 |
| molecular_function (GO:0003674) | 4888 | 956 | 659.4 | 1.45 | 1.22E-36 |
| DNA-binding transcription factor activity, RNA polymerase II-specific (GO:0000981) | 420 | 81 | 56.65 | 1.43 | 4.38E-02 |
| RNA polymerase II transcription regulatory region sequence-specific DNA binding (GO:0000977) | 474 | 91 | 63.94 | 1.42 | 3.24E-02 |
| DNA-binding transcription factor activity (GO:0003700) | 466 | 89 | 62.86 | 1.42 | 3.93E-02 |
| catalytic activity, acting on a protein (GO:0140096) | 825 | 153 | 111.3 | 1.37 | 7.13E-03 |
| catalytic activity (GO:0003824) | 2486 | 429 | 335.3 | 1.28 | 1.51E-05 |

**Supplementary Table 4.** List of gene ontology terms significantly over or underrepresented in the most conserved gene set (n = 100 genes of which 82 had functional annotation). Comparison against the aligned gene set (#ref set) in Panther GO-slim (n=1863). The table contains number of observed (# obs) and expected (# exp) occurrences of the GO-term in the most conserved gene set. Over- or underrepresentation is expressed as positive or negative fold change (fold). pFDR is p-value after FDR correction.

| **PANTHER GO-Slim Cellular Component** | **#ref set** | **#obs** | **#exp** | **fold** | **pFDR** |
| --- | --- | --- | --- | --- | --- |
| proteasome regulatory particle, base subcomplex (GO:0008540) | 7 | 4 | 0.31 | 12.98 | 2.18E-02 |
| cytosolic small ribosomal subunit (GO:0022627) | 16 | 8 | 0.7 | 11.36 | 2.97E-04 |
| proteasome accessory complex (GO:0022624) | 9 | 4 | 0.4 | 10.1 | 3.87E-02 |
| proteasome regulatory particle (GO:0005838) | 9 | 4 | 0.4 | 10.1 | 3.65E-02 |
| small ribosomal subunit (GO:0015935) | 26 | 10 | 1.14 | 8.74 | 2.10E-04 |
| endopeptidase complex (GO:1905369) | 17 | 6 | 0.75 | 8.02 | 1.16E-02 |
| proteasome complex (GO:0000502) | 17 | 6 | 0.75 | 8.02 | 1.05E-02 |
| spliceosomal complex (GO:0005681) | 19 | 6 | 0.84 | 7.17 | 1.56E-02 |
| Sm-like protein family complex (GO:0120114) | 16 | 5 | 0.7 | 7.1 | 3.71E-02 |
| peptidase complex (GO:1905368) | 23 | 7 | 1.01 | 6.91 | 8.38E-03 |
| cytosolic ribosome (GO:0022626) | 44 | 11 | 1.94 | 5.68 | 6.62E-04 |
| ribosomal subunit (GO:0044391) | 62 | 13 | 2.73 | 4.76 | 6.15E-04 |
| ribonucleoprotein complex (GO:1990904) | 106 | 21 | 4.67 | 4.5 | 6.48E-06 |
| ribosome (GO:0005840) | 67 | 13 | 2.95 | 4.41 | 9.21E-04 |
| cytosol (GO:0005829) | 126 | 14 | 5.55 | 2.52 | 3.54E-02 |
| protein-containing complex (GO:0032991) | 462 | 41 | 20.33 | 2.02 | 2.47E-04 |
| intracellular organelle (GO:0043229) | 742 | 49 | 32.66 | 1.5 | 1.63E-02 |
| organelle (GO:0043226) | 751 | 49 | 33.06 | 1.48 | 1.66E-02 |
|  |  |  |  |  |  |
| **PANTHER GO-Slim Molecular Function** | **#ref set** | **#obs** | **#exp** | **fold** | **pFDR** |
| structural constituent of ribosome (GO:0003735) | 65 | 12 | 2.86 | 4.19 | 2.49E-02 |
| structural molecule activity (GO:0005198) | 72 | 12 | 3.17 | 3.79 | 3.01E-02 |

**Supplementary Table 5.** Mean and standard deviation (Sd) for estimates of ω, *d_N_* and *d_S_* in the different lineages from two different methods, Codeml (PAML), and mapNH in Bio++ (mNH).

| **Branch** | **ω PAML** | **Sd** | **ω mNH** | **Sd** | **d_N_ PAML** | **Sd** | **d_N_ mNH** | **Sd** | **d_S_ PAML** | **Sd** | **d_S_ mNH** | **Sd** |
| --- | --- | --- | --- | --- | --- | --- | --- | --- | --- | --- | --- | --- |
| *B. mori* | 4.10E-02 | 4.95E-02 | 1.09E-01 | 6.73E-02 | 6.70E-02 | 4.96E-02 | 3.51E-02 | 2.18E-02 | 2.14E+00 | 1.73E+00 | 3.24E-01 | 3.78E-02 |
| *P. machaon* | 4.11E-02 | 4.20E-02 | 1.05E-01 | 7.25E-02 | 5.55E-02 | 5.30E-02 | 5.95E-02 | 4.31E-02 | 1.71E+00 | 1.61E+00 | 5.59E-01 | 6.73E-02 |
| *L. accius* | 4.22E-02 | 4.53E-02 | 1.01E-01 | 7.20E-02 | 5.34E-02 | 5.00E-02 | 5.86E-02 | 4.42E-02 | 1.56E+00 | 1.26E+00 | 5.68E-01 | 7.77E-02 |
| *L. sinapis* | 4.78E-02 | 1.07E-01 | 1.04E-01 | 7.11E-02 | 5.92E-02 | 5.32E-02 | 6.11E-02 | 4.50E-02 | 1.60E+00 | 1.24E+00 | 5.83E-01 | 5.84E-02 |
| *P. sennae* | 3.50E-02 | 5.62E-02 | 8.61E-02 | 6.47E-02 | 3.48E-02 | 3.59E-02 | 3.90E-02 | 3.09E-02 | 1.28E+00 | 1.05E+00 | 4.46E-01 | 6.35E-02 |
| *C. cecrops* | 4.74E-02 | 4.64E-02 | 1.17E-01 | 7.63E-02 | 7.00E-02 | 5.79E-02 | 7.34E-02 | 5.00E-02 | 1.86E+00 | 1.58E+00 | 6.20E-01 | 8.19E-02 |
| *D. plexippus* | 3.76E-02 | 6.74E-02 | 9.18E-02 | 6.34E-02 | 4.93E-02 | 4.62E-02 | 5.23E-02 | 3.85E-02 | 1.66E+00 | 1.22E+00 | 5.60E-01 | 6.21E-02 |
| *H. melpomene* | 3.82E-02 | 9.77E-02 | 8.41E-02 | 6.16E-02 | 4.22E-02 | 3.99E-02 | 4.55E-02 | 3.47E-02 | 1.47E+00 | 9.93E-01 | 5.35E-01 | 6.81E-02 |

**Supplementary Table 6.** Estimates and confidence intervals (CI) from a generalised linear model with d_N_/d_S_ as estimated in the branch model in PaML (codeml) as dependant value with species as fixed effect and gene ID as random effect variables.

|  | **d_N_/d_S_ (codeml)** | | | **d_N_/d_S_ (mapNH)** | | |
| --- | --- | --- | --- | --- | --- | --- |
| *Predictors* | *Estimates* | *CI* | *p* | *Estimates* | *CI* | *p* |
| (Intercept) | 0.03 | 0.03 – 0.03 | **<0.001** | 0.08 | 0.08 – 0.08 | **<0.001** |
| branch [P machaon] | 1.00 | 0.96 – 1.03 | 0.810 | 0.93 | 0.91 – 0.95 | **<0.001** |
| branch [L accius] | 1.01 | 0.97 – 1.05 | 0.640 | 0.89 | 0.87 – 0.91 | **<0.001** |
| branch [L sinapis] | 1.11 | 1.07 – 1.16 | **<0.001** | 0.95 | 0.93 – 0.97 | **<0.001** |
| branch [P sennae] | 0.80 | 0.77 – 0.83 | **<0.001** | 0.74 | 0.72 – 0.75 | **<0.001** |
| branch [C cecrops] | 1.16 | 1.12 – 1.20 | **<0.001** | 1.06 | 1.04 – 1.08 | **<0.001** |
| branch [D plexippus] | 0.88 | 0.85 – 0.91 | **<0.001** | 0.81 | 0.79 – 0.83 | **<0.001** |
| branch [H melpomene] | 0.85 | 0.81 – 0.88 | **<0.001** | 0.73 | 0.71 – 0.74 | **<0.001** |
| Observations | 21568 | | | 19560 | | |
| Marginal R^2^ / Conditional R^2^ | 0.017 / 0.420 | | | 0.039 / 0.538 | | |

**Supplementary Table 7.** Lineage-specific difference in mean ω, *d_N_* and *d_S_* between a) global and GC-conservative substitutions and b) between S -> W and W -> S. The significance of the difference between the substitution types were tested with paired Wilcoxon signed rank test with continuity correction. The statistic V and p-values are presented, p-values > 0.05 after correction for multiple testing are grey.

a) All versus GC-cons

| **Lineage** | **Diff ω** | **V** | **p-value** | **Diff *d_N_*** | **V** | **p-value** | **Diff *d_S_*** | **V** | **p-value** |
| --- | --- | --- | --- | --- | --- | --- | --- | --- | --- |
| ***B. mori*** | 0.0045 | 2520759 | 2.20E-16 | 0.00005 | 3059549 | 9.94E-02 | -0.00929 | 3848632 | 2.20E-16 |
| ***C. cecrops*** | 0.0058 | 2384153 | 2.20E-16 | -0.00008 | 3091590 | 2.61E-01 | -0.02861 | 4252358 | 2.20E-16 |
| ***D. plexippus*** | 0.0014 | 2866300 | 1.55E-06 | 0.00005 | 3082788 | 2.05E-01 | -0.00772 | 3450948 | 2.06E-06 |
| ***H. melpomene*** | -0.0002 | 3058207 | 9.50E-02 | 0.00047 | 2935248 | 2.34E-04 | 0.00861 | 2736281 | 4.21E-12 |
| ***L. accius*** | 0.0025 | 2730309 | 2.10E-12 | -0.00053 | 3229744 | 2.57E-01 | -0.01751 | 3892177 | 2.20E-16 |
| ***L. sinapis*** | 0.0013 | 2868324 | 1.82E-06 | -0.00082 | 3320765 | 8.78E-03 | -0.01576 | 3812941 | 2.20E-16 |
| ***P. machaon*** | 0.0020 | 2834514 | 1.01E-07 | -0.00088 | 3351959 | 1.75E-03 | -0.01648 | 3848619 | 2.20E-16 |
| ***P. sennae*** | 0.0025 | 2719755 | 6.03E-13 | -0.00040 | 3245499 | 1.64E-01 | -0.01877 | 4031890 | 2.20E-16 |

b) S->W versus W -> S

| **Lineage** | **Diff ω** | **V** | **p-value** | **Diff *d_N_*** | **V** | **p-value** | **Diff *d_S_*** | **V** | **p-value** |
| --- | --- | --- | --- | --- | --- | --- | --- | --- | --- |
| ***B. mori*** | -0.0089 | 2507067 | 2.20E-16 | 0.0084 | 4933835 | 2.20E-16 | 0.1226 | 5078349 | 2.20E-16 |
| ***C. cecrops*** | 0.0844 | 5210242 | 2.20E-16 | 0.0037 | 3727705 | 2.20E-16 | -0.3456 | 1721829 | 2.20E-16 |
| ***D. plexippus*** | 0.0251 | 4445044 | 2.20E-16 | 0.0102 | 4680006 | 2.20E-16 | -0.0134 | 3342260 | 2.96E-03 |
| ***H. melpomene*** | -0.0247 | 1706415 | 2.20E-16 | 0.0169 | 5326993 | 2.20E-16 | 0.3473 | 6045816 | 2.20E-16 |
| ***L. accius*** | 0.0293 | 4352199 | 2.20E-16 | 0.0015 | 3437248 | 6.08E-06 | -0.1450 | 2015393 | 2.20E-16 |
| ***L. sinapis*** | 0.0055 | 3371951 | 5.47E-04 | 0.0153 | 5056461 | 2.20E-16 | 0.1356 | 4830448 | 2.20E-16 |
| ***P. machaon*** | 0.0063 | 3328771 | 5.94E-03 | 0.0077 | 4298864 | 2.20E-16 | 0.0403 | 3929741 | 2.20E-16 |
| ***P. sennae*** | 0.0194 | 3998965 | 2.20E-16 | 0.0044 | 3972316 | 2.20E-16 | -0.0389 | 2928130 | 1.48E-04 |

**Supplementary Table 8.** Correlation between GC-content at third codon positions and estimated non-synonymous (*d_N_*), synonymous (*d_S_*) and ω in different substitution categories, all substitutions (All), only GC-conservative (GC-cons), strong to weak (S -> W) and weak to strong substitutions (S -> W). Font colour indicate p-value < 0.05 (black), p-value > 0.05 (grey). Heatmap colours indicate strength and direction of the correlation, positive (red) or negative (blue).

| **Branch** | **Subst. class** | ***d_N_*** | ***d_S_*** | **ω** |
| --- | --- | --- | --- | --- |
| *B. mori* | All | -0.11 | -0.20 | -0.06 |
| *C. cecrops* | All | 0.03 | -0.30 | 0.10 |
| *D. plexippus* | All | -0.09 | -0.16 | -0.07 |
| *H. melpomene* | All | -0.24 | 0.06 | -0.25 |
| *L. accius* | All | -0.20 | -0.45 | -0.11 |
| *L. sinapis* | All | -0.14 | -0.17 | -0.11 |
| *P. machaon* | All | -0.16 | -0.30 | -0.11 |
| *P. sennae* | All | -0.18 | -0.34 | -0.11 |
| *B. mori* | GC-cons | -0.09 | -0.01 | -0.09 |
| *C. cecrops* | GC-cons | 0.03 | -0.07 | 0.06 |
| *D. plexippus* | GC-cons | -0.06 | 0.01 | -0.07 |
| *H. melpomene* | GC-cons | -0.22 | -0.12 | -0.20 |
| *L. accius* | GC-cons | -0.17 | -0.22 | -0.13 |
| *L. sinapis* | GC-cons | -0.12 | -0.03 | -0.12 |
| *P. machaon* | GC-cons | -0.14 | -0.13 | -0.12 |
| *P. sennae* | GC-cons | -0.14 | -0.10 | -0.12 |
| *B. mori* | S -> W | -0.18 | -0.72 | 0.17 |
| *C. cecrops* | S -> W | -0.13 | -0.88 | 0.41 |
| *D. plexippus* | S -> W | -0.16 | -0.61 | 0.08 |
| *H. melpomene* | S -> W | -0.27 | -0.54 | -0.10 |
| *L. accius* | S -> W | -0.26 | -0.74 | 0.06 |
| *L. sinapis* | S -> W | -0.20 | -0.69 | 0.06 |
| *P. machaon* | S -> W | -0.28 | -0.76 | 0.07 |
| *P. sennae* | S -> W | -0.22 | -0.72 | 0.09 |
| *B. mori* | W -> S | -0.05 | 0.63 | -0.34 |
| *C. cecrops* | W -> S | 0.15 | 0.82 | -0.34 |
| *D. plexippus* | W -> S | -0.05 | 0.52 | -0.25 |
| *H. melpomene* | W -> S | -0.21 | 0.30 | -0.30 |
| *L. accius* | W -> S | -0.14 | 0.62 | -0.35 |
| *L. sinapis* | W -> S | -0.08 | 0.52 | -0.27 |
| *P. machaon* | W -> S | -0.06 | 0.63 | -0.33 |
| *P. sennae* | W -> S | -0.15 | 0.60 | -0.35 |

**Supplementary Table 9.** Median and standard deviation for gene-wise S -> W / W -> S-ratios for synonymous (Ratio*_dS_*) and non-synonymous substitution rates (Ratio*_dN_*) estimated per branch.

| **Branch** | **Ratio_dS_** | **sd_dS_** | **Ratio_dN_** | **sd_dN_** |
| --- | --- | --- | --- | --- |
| *B. mori* | 1.67 | 1.13 | 1.31 | 2.78 |
| *P. machaon* | 1.23 | 0.81 | 1.21 | 3.73 |
| *L. accius* | 0.87 | 0.50 | 1.04 | 2.89 |
| *L. sinapis* | 1.36 | 0.67 | 1.33 | 2.86 |
| *P. sennae* | 0.99 | 0.66 | 1.14 | 5.77 |
| *C. cecrops* | 0.72 | 0.74 | 1.12 | 2.74 |
| *D. plexippus* | 1.05 | 0.61 | 1.26 | 4.24 |
| *H. melpomene* | 1.98 | 1.04 | 1.45 | 4.86 |

**Supplementary Table 10.** Codon usage frequencies calculated as number of times the codon appeared per 1,000 codons a) in each lineage for CDS and aligned gene sets. (in separate file). b) Across lineages in the aligned gene set (Aligned) and for the 100 most conserved genes (Conserved).

a) In sep file

b)

| Codon | Aligned | Conserved |
| --- | --- | --- |
| GCA | 17.0 | 16.2 |
| GCC | 15.6 | 15.0 |
| GCG | 15.6 | 11.3 |
| GCT | 21.0 | 21.6 |
| TGC | 9.5 | 9.2 |
| TGT | 10.7 | 9.9 |
| GAC | 24.9 | 25.8 |
| GAT | 30.0 | 35.1 |
| GAA | 38.3 | 42.1 |
| GAG | 27.0 | 29.9 |
| TTC | 20.1 | 20.1 |
| TTT | 18.4 | 19.3 |
| GGA | 16.9 | 19.3 |
| GGC | 15.9 | 16.7 |
| GGG | 7.8 | 6.8 |
| GGT | 16.5 | 22.5 |
| CAC | 12.1 | 11.4 |
| CAT | 12.3 | 11.0 |
| ATA | 22.2 | 19.2 |
| ATC | 14.9 | 17.4 |
| ATT | 19.1 | 22.0 |
| AAA | 38.0 | 40.2 |
| AAG | 28.0 | 37.4 |
| CTA | 11.1 | 9.4 |
| CTC | 13.2 | 12.9 |
| CTG | 19.7 | 16.8 |
| CTT | 12.4 | 14.3 |
| TTA | 19.4 | 16.9 |
| TTG | 18.1 | 16.4 |
| ATG | 24.3 | 27.7 |
| AAC | 20.9 | 19.4 |
| AAT | 25.8 | 21.2 |
| CCA | 15.9 | 14.2 |
| CCC | 10.2 | 9.7 |
| CCG | 11.4 | 8.3 |
| CCT | 13.4 | 12.2 |
| CAA | 20.5 | 21.3 |
| CAG | 17.5 | 18.5 |
| AGA | 16.6 | 19.6 |
| AGG | 9.6 | 10.5 |
| CGA | 7.3 | 7.4 |
| CGC | 8.9 | 9.9 |
| CGG | 5.8 | 5.4 |
| CGT | 7.5 | 12.2 |
| AGC | 11.1 | 7.1 |
| AGT | 13.3 | 8.9 |
| TCA | 14.6 | 13.3 |
| TCC | 10.1 | 9.4 |
| TCG | 9.5 | 7.0 |
| TCT | 13.6 | 12.4 |
| ACA | 18.5 | 19.0 |
| ACC | 10.8 | 9.9 |
| ACG | 10.4 | 7.2 |
| ACT | 15.5 | 15.2 |
| GTA | 14.1 | 14.8 |
| GTC | 12.4 | 12.7 |
| GTG | 21.7 | 20.9 |
| GTT | 17.2 | 18.4 |
| TGG | 12.2 | 9.0 |
| TAC | 17.4 | 15.8 |
| TAT | 16.4 | 15.6 |

**Supplementary Table 11.** a) Relative synonymous codon usage (RSCU) in the aligned gene set per branch. b) Number of A,T,G and C-ending codons per branch with RSCU above 1, corresponding to increased relative usage. Total number of codons for all species (8 x 61).

a)

| Codon | AA | B. mori | C. cecrops | D. plexippus | | H. melpomene | L. accius | L. sinapis | P. machaon | P. sennae |
| --- | --- | --- | --- | --- | --- | --- | --- | --- | --- | --- |
| AAA | K | 1.19 | 1.08 | 1.16 | 1.32 | | 1.09 | 1.16 | 1.16 | 1.13 |
| AAC | N | 0.97 | 1.02 | 0.93 | 0.74 | | 0.94 | 0.83 | 0.88 | 0.88 |
| AAG | K | 0.81 | 0.92 | 0.84 | 0.68 | | 0.91 | 0.84 | 0.84 | 0.87 |
| AAT | N | 1.03 | 0.98 | 1.07 | 1.26 | | 1.06 | 1.17 | 1.12 | 1.12 |
| ACA | T | 1.18 | 1.18 | 1.30 | 1.42 | | 1.17 | 1.51 | 1.40 | 1.28 |
| ACC | T | 0.84 | 0.90 | 0.84 | 0.61 | | 0.81 | 0.70 | 0.73 | 0.78 |
| ACG | T | 0.90 | 0.96 | 0.78 | 0.66 | | 0.83 | 0.66 | 0.75 | 0.86 |
| ACT | T | 1.08 | 0.96 | 1.08 | 1.31 | | 1.19 | 1.13 | 1.12 | 1.08 |
| AGA | R | 1.66 | 1.52 | 1.86 | 1.92 | | 1.58 | 1.88 | 1.65 | 1.65 |
| AGC | S | 0.97 | 1.06 | 0.89 | 0.76 | | 0.92 | 0.84 | 0.89 | 0.91 |
| AGG | R | 1.04 | 1.02 | 1.11 | 0.92 | | 0.94 | 0.99 | 0.84 | 1.07 |
| AGT | S | 1.01 | 0.96 | 1.07 | 1.21 | | 1.09 | 1.18 | 1.12 | 1.09 |
| ATA | I | 1.17 | 1.23 | 1.30 | 1.38 | | 1.10 | 1.19 | 1.21 | 1.14 |
| ATC | I | 0.89 | 0.88 | 0.79 | 0.55 | | 0.92 | 0.71 | 0.81 | 0.85 |
| ATT | I | 0.96 | 0.92 | 0.94 | 1.10 | | 1.01 | 1.13 | 1.01 | 1.05 |
| CAA | Q | 1.06 | 0.95 | 1.04 | 1.29 | | 1.01 | 1.11 | 1.05 | 1.13 |
| CAC | H | 1.09 | 1.17 | 1.00 | 0.89 | | 0.98 | 0.93 | 0.98 | 1.00 |
| CAG | Q | 0.94 | 1.05 | 0.96 | 0.71 | | 0.99 | 0.89 | 0.95 | 0.87 |
| CAT | H | 0.91 | 0.83 | 1.00 | 1.11 | | 1.02 | 1.07 | 1.02 | 1.00 |
| CCA | P | 1.10 | 1.10 | 1.23 | 1.36 | | 1.17 | 1.46 | 1.17 | 1.24 |
| CCC | P | 0.80 | 0.93 | 0.81 | 0.70 | | 0.78 | 0.71 | 0.82 | 0.86 |
| CCG | P | 1.07 | 1.10 | 0.96 | 0.73 | | 0.99 | 0.79 | 0.97 | 0.89 |
| CCT | P | 1.03 | 0.87 | 1.01 | 1.20 | | 1.06 | 1.05 | 1.04 | 1.01 |
| CGA | R | 0.79 | 0.66 | 0.74 | 0.78 | | 0.88 | 0.85 | 0.75 | 0.78 |
| CGC | R | 0.95 | 1.28 | 0.75 | 0.91 | | 1.01 | 0.78 | 1.18 | 1.05 |
| CGG | R | 0.64 | 0.81 | 0.54 | 0.46 | | 0.76 | 0.66 | 0.60 | 0.64 |
| CGT | R | 0.80 | 0.60 | 0.89 | 0.89 | | 0.72 | 0.72 | 0.87 | 0.69 |
| CTA | L | 0.69 | 0.64 | 0.68 | 0.79 | | 0.71 | 0.70 | 0.66 | 0.65 |
| CTC | L | 0.89 | 0.92 | 0.87 | 0.68 | | 0.88 | 0.71 | 0.85 | 0.88 |
| CTG | L | 1.31 | 1.70 | 1.26 | 0.84 | | 1.32 | 1.23 | 1.30 | 1.21 |
| CTT | L | 0.72 | 0.62 | 0.78 | 0.84 | | 0.72 | 0.86 | 0.74 | 0.74 |
| GAA | E | 1.19 | 1.00 | 1.17 | 1.33 | | 1.12 | 1.19 | 1.14 | 1.19 |
| GAC | D | 0.97 | 1.08 | 0.92 | 0.80 | | 0.97 | 0.82 | 0.89 | 0.89 |
| GAG | E | 0.81 | 1.00 | 0.83 | 0.67 | | 0.88 | 0.81 | 0.86 | 0.81 |
| GAT | D | 1.03 | 0.92 | 1.08 | 1.20 | | 1.03 | 1.18 | 1.11 | 1.11 |
| GCA | A | 0.88 | 0.82 | 0.85 | 1.04 | | 0.92 | 1.14 | 1.00 | 0.94 |
| GCC | A | 1.04 | 1.02 | 0.97 | 0.80 | | 0.93 | 0.81 | 0.84 | 0.87 |
| GCG | A | 0.93 | 1.20 | 0.84 | 0.88 | | 0.98 | 0.78 | 1.06 | 1.03 |
| GCT | A | 1.16 | 0.95 | 1.34 | 1.28 | | 1.18 | 1.28 | 1.10 | 1.16 |
| GGA | G | 1.22 | 1.04 | 1.20 | 1.21 | | 1.16 | 1.26 | 1.06 | 1.13 |
| GGC | G | 1.14 | 1.39 | 1.00 | 1.04 | | 1.18 | 1.00 | 1.22 | 1.18 |
| GGG | G | 0.56 | 0.64 | 0.55 | 0.50 | | 0.64 | 0.54 | 0.48 | 0.60 |
| GGT | G | 1.09 | 0.93 | 1.26 | 1.26 | | 1.02 | 1.21 | 1.24 | 1.09 |
| GTA | V | 0.80 | 0.76 | 0.78 | 1.05 | | 0.84 | 0.86 | 0.86 | 0.84 |
| GTC | V | 0.92 | 0.80 | 0.85 | 0.65 | | 0.80 | 0.68 | 0.74 | 0.72 |
| GTG | V | 1.26 | 1.62 | 1.27 | 1.17 | | 1.42 | 1.28 | 1.37 | 1.44 |
| GTT | V | 1.03 | 0.82 | 1.10 | 1.13 | | 0.94 | 1.18 | 1.02 | 1.01 |
| TAC | Y | 1.12 | 1.17 | 1.03 | 0.86 | | 1.11 | 0.96 | 1.07 | 1.01 |
| TAT | Y | 0.88 | 0.83 | 0.97 | 1.14 | | 0.89 | 1.04 | 0.93 | 0.99 |
| TCA | S | 1.08 | 0.98 | 1.19 | 1.28 | | 1.08 | 1.32 | 1.19 | 1.15 |
| TCC | S | 0.81 | 0.95 | 0.91 | 0.71 | | 0.82 | 0.70 | 0.79 | 0.86 |
| TCG | S | 0.96 | 0.95 | 0.76 | 0.66 | | 0.86 | 0.70 | 0.77 | 0.81 |
| TCT | S | 1.05 | 0.98 | 1.06 | 1.25 | | 1.11 | 1.14 | 1.11 | 1.06 |
| TGC | C | 1.05 | 1.09 | 0.84 | 0.85 | | 0.95 | 0.85 | 1.00 | 0.97 |
| TGT | C | 0.95 | 0.91 | 1.16 | 1.15 | | 1.05 | 1.15 | 1.00 | 1.03 |
| TTA | L | 1.12 | 1.02 | 1.16 | 1.59 | | 1.09 | 1.22 | 1.21 | 1.24 |
| TTC | F | 1.13 | 1.15 | 1.10 | 0.88 | | 1.10 | 0.95 | 1.07 | 1.07 |
| TTG | L | 1.16 | 0.98 | 1.14 | 1.15 | | 1.16 | 1.16 | 1.12 | 1.17 |
| TTT | F | 0.87 | 0.85 | 0.90 | 1.12 | | 0.90 | 1.05 | 0.93 | 0.93 |

b)

|  | A | T | G | C |
| --- | --- | --- | --- | --- |
| *B. mori* | 10 | 9 | 5 | 6 |
| *C. cecrops* | 7 | 0 | 7 | 10 |
| *D. plexippus* | 10 | 10 | 4 | 3 |
| *H. melpomene* | 12 | 14 | 2 | 1 |
| *L. accius* | 10 | 11 | 3 | 4 |
| *L. sinapis* | 11 | 14 | 3 | 0 |
| *P. machaon* | 10 | 12 | 4 | 4 |
| *P. sennae* | 10 | 12 | 5 | 4 |
| Nr codons RSCU>1 | 80 | 82 | 33 | 32 |
| Total nr of codons | 112 | 128 | 104 | 128 |
| Average percent overrepresented (%) | 71.4 | 64.1 | 31.8 | 25.0 |

**Supplementary Table 12.** Characterisation of tRNA-genes in *L. sinapis*. a) Summary of observed specific anticodons (Obs_unique), versus the expected number per third codon position nucleotide when all codons were used (Exp_unique). Total number of anticodons per third position nucleotide (Tot_count). b) Proportion of nucleotides and GC-content (w/o T^ACC^) in tRNA-gene codons at first, second and third codon positions. c) Table of tRNA-anticodon count. Number of synonymous codons (Syn_nr), presence or absence of specific anticodon in the tRNA-gene set (Presence_trna), observed count of specific tRNA-genes (Obs_count_trna), expected count of each codon based on total count of tRNA-genes per amino acid (Exp_count_trna), observed (Obs_fr_trna) and expected (Exp_fr_trna) fraction and relative abundance of isoacceptor tRNA-anticodons (RAIT_trna). For the coding sequences relative synonymous codon usage (RSCU_cds), total count of codons (Count_cds), number of codons per 1000 codons (Frequency_cds) and the observed fraction of codons (Fraction_cds) are given.

a)

| **Counts** | **A** | **T** | **G** | **C** | **Total** |
| --- | --- | --- | --- | --- | --- |
| Obs_unique | 14 | 10 | 14 | 12 | 50 |
| Exp_unique | 14 | 16 | 15 | 16 | 61 |
| Tot_count | 103 | 91 | 94 | 173 | 461 |

b)

| **%** | **A** | **T** | **G** | **C** | **GC** | **GC excl T^ACC^** |
| --- | --- | --- | --- | --- | --- | --- |
| First | 35.36 | 17.35 | 27.77 | 19.52 | 47.29 | 53.96 |
| Second | 28.42 | 21.04 | 16.92 | 33.62 | 50.54 | 43.56 |
| Third | 22.34 | 19.74 | 20.39 | 37.53 | 57.92 | 51.98 |

c)

| **Codon** | **AA** | **Syn nr** | **Presence trna** | **Obs count trna** | **Exp count trna** | **Obs fr trna** | **Exp fr trna** | **RSCU trna** | **RSCU cds** | **Count cds** | **Frequency cds** | **Fraction cds** |
| --- | --- | --- | --- | --- | --- | --- | --- | --- | --- | --- | --- | --- |
| GCA | A | 4 | 1 | 7 | 9.50 | 0.18 | 0.25 | 0.74 | 1.08 | 142706 | 17.61 | 0.27 |
| GCC | A | 4 | 1 | 2 | 9.50 | 0.05 | 0.25 | 0.21 | 0.84 | 111626 | 13.78 | 0.21 |
| GCG | A | 4 | 1 | 6 | 9.50 | 0.16 | 0.25 | 0.63 | 0.87 | 114647 | 14.15 | 0.22 |
| GCT | A | 4 | 1 | 23 | 9.50 | 0.61 | 0.25 | 2.42 | 1.21 | 160396 | 19.79 | 0.30 |
| TGC | C | 2 | 1 | 9 | 5.00 | 0.90 | 0.50 | 1.80 | 0.86 | 78435 | 9.68 | 0.43 |
| TGT | C | 2 | 1 | 1 | 5.00 | 0.10 | 0.50 | 0.20 | 1.14 | 103573 | 12.78 | 0.57 |
| GAC | D | 2 | 1 | 20 | 10.00 | 1.00 | 0.50 | 2.00 | 0.86 | 191637 | 23.65 | 0.43 |
| GAT | D | 2 | 0 | 0 | 10.00 | 0.00 | 0.50 | 0.00 | 1.14 | 254396 | 31.39 | 0.57 |
| GAA | E | 2 | 1 | 14 | 12.00 | 0.58 | 0.50 | 1.17 | 1.18 | 313312 | 38.67 | 0.59 |
| GAG | E | 2 | 1 | 10 | 12.00 | 0.42 | 0.50 | 0.83 | 0.82 | 218127 | 26.92 | 0.41 |
| TTC | F | 2 | 1 | 8 | 4.00 | 1.00 | 0.50 | 2.00 | 0.97 | 141274 | 17.43 | 0.48 |
| TTT | F | 2 | 0 | 0 | 4.00 | 0.00 | 0.50 | 0.00 | 1.03 | 150848 | 18.62 | 0.52 |
| GGA | G | 4 | 1 | 10 | 6.25 | 0.40 | 0.25 | 1.60 | 1.26 | 139617 | 17.23 | 0.32 |
| GGC | G | 4 | 1 | 12 | 6.25 | 0.48 | 0.25 | 1.92 | 1.04 | 115501 | 14.25 | 0.26 |
| GGG | G | 4 | 1 | 2 | 6.25 | 0.08 | 0.25 | 0.32 | 0.55 | 60659 | 7.49 | 0.14 |
| GGT | G | 4 | 1 | 1 | 6.25 | 0.04 | 0.25 | 0.16 | 1.15 | 127393 | 15.72 | 0.29 |
| CAC | H | 2 | 1 | 24 | 12.00 | 1.00 | 0.50 | 2.00 | 0.98 | 97538 | 12.04 | 0.49 |
| CAT | H | 2 | 0 | 0 | 12.00 | 0.00 | 0.50 | 0.00 | 1.02 | 102222 | 12.62 | 0.51 |
| ATA | I | 3 | 1 | 4 | 5.28 | 0.25 | 0.33 | 0.76 | 1.17 | 178278 | 22.00 | 0.39 |
| ATC | I | 3 | 1 | 1 | 5.28 | 0.06 | 0.33 | 0.19 | 0.74 | 112645 | 13.90 | 0.25 |
| ATT | I | 3 | 1 | 11 | 5.28 | 0.69 | 0.33 | 2.08 | 1.12 | 169363 | 20.90 | 0.37 |
| AAA | K | 2 | 1 | 10 | 10.00 | 0.50 | 0.50 | 1.00 | 1.17 | 304539 | 37.58 | 0.58 |
| AAG | K | 2 | 1 | 10 | 10.00 | 0.50 | 0.50 | 1.00 | 0.83 | 217411 | 26.83 | 0.42 |
| CTA | L | 6 | 1 | 3 | 5.95 | 0.09 | 0.17 | 0.51 | 0.69 | 85368 | 10.54 | 0.12 |
| CTC | L | 6 | 0 | 0 | 5.95 | 0.00 | 0.17 | 0.00 | 0.75 | 92457 | 11.41 | 0.13 |
| CTG | L | 6 | 1 | 4 | 5.95 | 0.11 | 0.17 | 0.67 | 1.28 | 159342 | 19.66 | 0.22 |
| CTT | L | 6 | 1 | 6 | 5.95 | 0.17 | 0.17 | 1.01 | 0.84 | 104531 | 12.90 | 0.14 |
| TTA | L | 6 | 1 | 17 | 5.95 | 0.49 | 0.17 | 2.86 | 1.19 | 147073 | 18.15 | 0.20 |
| TTG | L | 6 | 1 | 5 | 5.95 | 0.14 | 0.17 | 0.84 | 1.14 | 140624 | 17.35 | 0.19 |
| ATG | M | 1 | 1 | 17 | 17.00 | 1.00 | 1.00 | 1.00 | 1.00 | 183880 | 22.69 | 1.00 |
| AAC | N | 2 | 1 | 13 | 6.50 | 1.00 | 0.50 | 2.00 | 0.85 | 173097 | 21.36 | 0.42 |
| AAT | N | 2 | 0 | 0 | 6.50 | 0.00 | 0.50 | 0.00 | 1.15 | 235912 | 29.11 | 0.58 |
| CCA | P | 4 | 1 | 7 | 4.75 | 0.37 | 0.25 | 1.47 | 1.39 | 147376 | 18.19 | 0.35 |
| CCC | P | 4 | 0 | 0 | 4.75 | 0.00 | 0.25 | 0.00 | 0.73 | 77321 | 9.54 | 0.18 |
| CCG | P | 4 | 1 | 3 | 4.75 | 0.16 | 0.25 | 0.63 | 0.85 | 90205 | 11.13 | 0.21 |
| CCT | P | 4 | 1 | 9 | 4.75 | 0.47 | 0.25 | 1.90 | 1.02 | 108125 | 13.34 | 0.26 |
| CAA | Q | 2 | 1 | 8 | 6.50 | 0.62 | 0.50 | 1.23 | 1.10 | 173282 | 21.38 | 0.55 |
| CAG | Q | 2 | 1 | 5 | 6.50 | 0.39 | 0.50 | 0.77 | 0.90 | 142690 | 17.61 | 0.45 |
| AGA | R | 6 | 1 | 3 | 4.93 | 0.10 | 0.17 | 0.61 | 1.75 | 135911 | 16.77 | 0.30 |
| AGG | R | 6 | 1 | 5 | 4.93 | 0.17 | 0.17 | 1.01 | 0.94 | 72882 | 8.99 | 0.16 |
| CGA | R | 6 | 1 | 5 | 4.93 | 0.17 | 0.17 | 1.01 | 0.86 | 66921 | 8.26 | 0.15 |
| CGC | R | 6 | 1 | 3 | 4.93 | 0.10 | 0.17 | 0.61 | 0.89 | 69005 | 8.52 | 0.15 |
| CGG | R | 6 | 0 | 0 | 4.93 | 0.00 | 0.17 | 0.00 | 0.69 | 53832 | 6.64 | 0.12 |
| CGT | R | 6 | 1 | 13 | 4.93 | 0.45 | 0.17 | 2.64 | 0.75 | 58239 | 7.19 | 0.13 |
| AGC | S | 6 | 1 | 7 | 3.91 | 0.30 | 0.17 | 1.79 | 0.87 | 93850 | 11.58 | 0.15 |
| AGT | S | 6 | 0 | 0 | 3.91 | 0.00 | 0.17 | 0.00 | 1.14 | 121969 | 15.05 | 0.19 |
| TCA | S | 6 | 1 | 6 | 3.91 | 0.26 | 0.17 | 1.54 | 1.28 | 137239 | 16.94 | 0.22 |
| TCC | S | 6 | 0 | 0 | 3.91 | 0.00 | 0.17 | 0.00 | 0.72 | 77892 | 9.61 | 0.12 |
| TCG | S | 6 | 1 | 3 | 3.91 | 0.13 | 0.17 | 0.76 | 0.77 | 82695 | 10.21 | 0.13 |
| TCT | S | 6 | 1 | 7 | 3.91 | 0.30 | 0.17 | 1.79 | 1.11 | 119269 | 14.72 | 0.19 |
| ACA | T | 4 | 1 | 4 | 20.50 | 0.05 | 0.25 | 0.20 | 1.43 | 165440 | 20.42 | 0.36 |
| ACC | T | 4 | 1 | 57 | 20.50 | 0.70 | 0.25 | 2.78 | 0.74 | 85041 | 10.50 | 0.18 |
| ACG | T | 4 | 1 | 10 | 20.50 | 0.12 | 0.25 | 0.49 | 0.73 | 84117 | 10.38 | 0.18 |
| ACT | T | 4 | 1 | 11 | 20.50 | 0.13 | 0.25 | 0.54 | 1.10 | 127758 | 15.77 | 0.28 |
| GTA | V | 4 | 1 | 5 | 5.25 | 0.24 | 0.25 | 0.95 | 0.86 | 109172 | 13.47 | 0.21 |
| GTC | V | 4 | 0 | 0 | 5.25 | 0.00 | 0.25 | 0.00 | 0.71 | 91092 | 11.24 | 0.18 |
| GTG | V | 4 | 1 | 7 | 5.25 | 0.33 | 0.25 | 1.33 | 1.29 | 164951 | 20.36 | 0.32 |
| GTT | V | 4 | 1 | 9 | 5.25 | 0.43 | 0.25 | 1.72 | 1.14 | 145297 | 17.93 | 0.29 |
| TGG | W | 1 | 1 | 7 | 7.00 | 1.00 | 1.00 | 1.00 | 1.00 | 90483 | 11.17 | 1.00 |
| TAC | Y | 2 | 1 | 17 | 8.50 | 1.00 | 0.50 | 2.00 | 0.98 | 129258 | 15.95 | 0.49 |
| TAT | Y | 2 | 0 | 0 | 8.50 | 0.00 | 0.50 | 0.00 | 1.02 | 133457 | 16.47 | 0.51 |

**Supplementary Table 13.** a) Branch-wise mean effective number of codons (ENC_obs_) and standard deviation (sd), expected ENC based on GC-content alone (ENC_exp_) and proportional difference between ENC and expected ENC (ENC_diff_). Lineage-specific Spearman’s rank correlation between b) ENCobs and c) ENCdiff and ω, *d_N_*, and *d_S_* with significant p-value after correction for multiple testing in bold.

a)

| Branch | ENC_obs_ | sd | ENC_exp_ | sd | ENC_diff_ | sd |
| --- | --- | --- | --- | --- | --- | --- |
| *B. mori* | 54.12 | 4.84 | 55.02 | 4.31 | 1.74E-02 | 4.87E-02 |
| *C. cecrops* | 53.23 | 5.06 | 54.30 | 4.74 | 2.47E-02 | 5.07E-02 |
| *D. plexippus* | 54.29 | 4.86 | 55.13 | 4.42 | 1.62E-02 | 4.96E-02 |
| *H. melpomene* | 53.57 | 4.97 | 54.49 | 4.40 | 2.01E-02 | 5.13E-02 |
| *L. accius* | 53.42 | 5.28 | 54.58 | 4.64 | 2.35E-02 | 5.30E-02 |
| *L. sinapis* | 51.86 | 5.72 | 52.84 | 5.63 | 2.10E-02 | 5.41E-02 |
| *P. machaon* | 54.04 | 4.45 | 55.32 | 3.93 | 2.46E-02 | 5.05E-02 |
| *P. sennae* | 52.75 | 5.33 | 53.46 | 5.08 | 1.79E-02 | 5.22E-02 |

b)

| **ENCobs** | **Lineage** | **rho_ω_** | **p-value** | **rho*_dN_*** | **p-value** | **rho*_dS_*** | **p-value** |
| --- | --- | --- | --- | --- | --- | --- | --- |
| Global | *B. mori* | -0.12 | **5.00E-10** | -0.06 | **1.31E-03** | 0.32 | **2.20E-16** |
| Global | *C. cecrops* | -0.10 | **6.68E-08** | -0.02 | 2.34E-01 | 0.35 | **2.20E-16** |
| Global | *D. plexippus* | -0.09 | **2.25E-06** | -0.06 | 2.15E-03 | 0.18 | **2.20E-16** |
| Global | *H. melpomene* | -0.20 | **2.20E-16** | -0.16 | **2.20E-16** | 0.23 | **2.20E-16** |
| Global | *L. accius* | -0.08 | **1.70E-05** | -0.02 | 3.09E-01 | 0.24 | **2.20E-16** |
| Global | *L. sinapis* | -0.08 | **6.98E-06** | -0.06 | 1.60E-03 | 0.15 | **4.67E-15** |
| Global | *P. machaon* | -0.12 | **7.89E-11** | -0.08 | **1.11E-05** | 0.17 | **2.20E-16** |
| Global | *P. sennae* | -0.08 | **4.18E-05** | -0.03 | 1.47E-01 | 0.22 | **2.20E-16** |
| GC-cons | *B. mori* | -0.08 | **5.06E-05** | -0.06 | **1.30E-03** | 0.06 | 2.28E-03 |
| GC-cons | *C. cecrops* | -0.06 | **8.08E-04** | -0.03 | 6.83E-02 | 0.13 | **3.79E-13** |
| GC-cons | *D. plexippus* | -0.07 | **3.92E-04** | -0.06 | 2.65E-03 | 0.05 | 6.48E-03 |
| GC-cons | *H. melpomene* | -0.16 | **2.20E-16** | -0.16 | **2.20E-16** | -0.01 | 5.69E-01 |
| GC-cons | *L. accius* | -0.05 | 9.07E-03 | -0.03 | 1.14E-01 | 0.07 | **2.08E-04** |
| GC-cons | *L. sinapis* | -0.06 | 2.51E-03 | -0.06 | 2.71E-03 | 0.01 | 4.45E-01 |
| GC-cons | *P. machaon* | -0.08 | **5.44E-06** | -0.07 | **6.15E-05** | 0.02 | 2.08E-01 |
| GC-cons | *P. sennae* | -0.05 | 5.39E-03 | -0.03 | 1.19E-01 | 0.10 | **3.63E-08** |
| S -> W | *B. mori* | -0.04 | 2.36E-02 | -0.05 | 1.48E-02 | -0.10 | **2.57E-07** |
| S -> W | *C. cecrops* | -0.10 | **2.28E-08** | 0.03 | 6.31E-02 | 0.16 | **2.20E-16** |
| S -> W | *D. plexippus* | -0.03 | 7.06E-02 | -0.03 | 1.18E-01 | -0.03 | 7.02E-02 |
| S -> W | *H. melpomene* | -0.07 | **6.10E-05** | -0.16 | **2.20E-16** | -0.30 | **2.20E-16** |
| S -> W | *L. accius* | -0.05 | 1.10E-02 | 0.01 | 7.66E-01 | 0.03 | 1.24E-01 |
| S -> W | *L. sinapis* | 0.02 | 2.94E-01 | -0.06 | **7.51E-04** | -0.30 | **2.20E-16** |
| S -> W | *P. machaon* | -0.04 | 3.07E-02 | -0.06 | **1.28E-03** | -0.14 | **5.58E-14** |
| S -> W | *P. sennae* | -0.01 | 4.87E-01 | -0.01 | 6.00E-01 | -0.07 | **2.98E-04** |
| W -> S | *B. mori* | -0.09 | **2.89E-06** | -0.06 | 2.25E-03 | 0.16 | **2.20E-16** |
| W -> S | *C. cecrops* | 0.06 | 2.18E-03 | -0.05 | 7.61E-03 | -0.12 | **6.38E-11** |
| W -> S | *D. plexippus* | -0.10 | **2.44E-07** | -0.08 | **2.92E-05** | 0.08 | **9.48E-06** |
| W -> S | *H. melpomene* | -0.21 | **2.20E-16** | -0.15 | **1.33E-15** | 0.23 | **2.20E-16** |
| W -> S | *L. accius* | -0.02 | 2.78E-01 | -0.03 | 1.76E-01 | 0.07 | **4.47E-04** |
| W -> S | *L. sinapis* | -0.13 | **5.65E-13** | -0.05 | 5.12E-03 | 0.27 | **2.20E-16** |
| W -> S | *P. machaon* | -0.11 | **1.13E-09** | -0.09 | **3.69E-06** | 0.16 | **2.20E-16** |
| W -> S | *P. sennae* | -0.07 | **1.86E-04** | -0.04 | 4.41E-02 | 0.14 | **1.23E-14** |

c)

| **ENCdiff** | **Lineage** | **rho_ω_** | **p-value** | **rho*_dN_*** | **p-value** | **rho*_dS_*** | **p-value** |
| --- | --- | --- | --- | --- | --- | --- | --- |
| Global | *B. mori* | 0.02 | 2.13E-01 | -0.01 | 7.27E-01 | -0.15 | **1.32E-15** |
| Global | *C. cecrops* | -0.04 | 3.99E-02 | -0.05 | 6.32E-03 | -0.09 | **2.92E-06** |
| Global | *D. plexippus* | 0.00 | 9.86E-01 | -0.01 | 7.51E-01 | -0.05 | 3.99E-03 |
| Global | *H. melpomene* | -0.06 | **5.09E-04** | -0.08 | **1.88E-05** | -0.12 | **2.00E-10** |
| Global | *L. accius* | -0.06 | **1.12E-03** | -0.07 | **9.88E-05** | -0.10 | **3.12E-07** |
| Global | *L. sinapis* | -0.06 | **1.35E-03** | -0.07 | **6.67E-05** | -0.11 | **7.07E-10** |
| Global | *P. machaon* | -0.07 | **1.56E-04** | -0.09 | **9.35E-07** | -0.12 | **2.99E-10** |
| Global | *P. sennae* | -0.05 | 1.31E-02 | -0.08 | **2.93E-05** | -0.19 | **2.20E-16** |
| GC-cons | *B. mori* | 0.00 | 9.49E-01 | -0.01 | 4.34E-01 | -0.08 | **2.19E-05** |
| GC-cons | *C. cecrops* | -0.02 | 2.52E-01 | -0.04 | 3.47E-02 | -0.10 | **4.17E-08** |
| GC-cons | *D. plexippus* | 0.01 | 6.06E-01 | 0.00 | 9.24E-01 | -0.06 | 2.67E-03 |
| GC-cons | *H. melpomene* | -0.05 | 1.29E-02 | -0.06 | **7.12E-04** | -0.10 | **4.56E-08** |
| GC-cons | *L. accius* | -0.04 | 1.62E-02 | -0.06 | **1.20E-03** | -0.10 | **2.26E-07** |
| GC-cons | *L. sinapis* | -0.07 | **3.74E-04** | -0.07 | **1.18E-04** | -0.06 | **6.65E-04** |
| GC-cons | *P. machaon* | -0.06 | 2.41E-03 | -0.08 | **1.16E-05** | -0.13 | **1.54E-12** |
| GC-cons | *P. sennae* | -0.04 | 3.15E-02 | -0.06 | **5.83E-04** | -0.13 | **8.22E-13** |
| S -> W | *B. mori* | 0.06 | 1.94E-03 | -0.01 | 4.22E-01 | -0.13 | **8.28E-13** |
| S -> W | *C. cecrops* | -0.02 | 1.99E-01 | -0.06 | **7.30E-04** | -0.05 | 4.35E-03 |
| S -> W | *D. plexippus* | 0.02 | 2.10E-01 | -0.03 | 9.42E-02 | -0.18 | **2.20E-16** |
| S -> W | *H. melpomene* | -0.07 | **1.23E-04** | -0.09 | **4.73E-07** | -0.07 | **2.24E-04** |
| S -> W | *L. accius* | -0.06 | 2.28E-03 | -0.07 | **1.08E-04** | -0.05 | 3.71E-03 |
| S -> W | *L. sinapis* | -0.06 | 2.39E-03 | -0.08 | **2.35E-05** | -0.05 | 9.32E-03 |
| S -> W | *P. machaon* | -0.07 | **2.15E-04** | -0.09 | **2.03E-06** | -0.05 | 3.87E-03 |
| S -> W | *P. sennae* | -0.01 | 6.02E-01 | -0.08 | **2.19E-05** | -0.15 | **2.20E-16** |
| W -> S | *B. mori* | -0.04 | 5.09E-02 | 0.01 | 6.81E-01 | 0.06 | **1.11E-03** |
| W -> S | *C. cecrops* | -0.05 | 8.48E-03 | -0.04 | 2.20E-02 | -0.01 | 4.95E-01 |
| W -> S | *D. plexippus* | -0.02 | 2.11E-01 | 0.02 | 3.24E-01 | 0.12 | **3.89E-11** |
| W -> S | *H. melpomene* | -0.06 | 2.93E-03 | -0.07 | **1.45E-04** | -0.05 | 3.31E-03 |
| W -> S | *L. accius* | -0.07 | **4.22E-04** | -0.07 | **9.53E-05** | -0.01 | 4.97E-01 |
| W -> S | *L. sinapis* | -0.05 | 1.48E-02 | -0.05 | 5.21E-03 | -0.05 | 1.45E-02 |
| W -> S | *P. machaon* | -0.07 | **3.25E-04** | -0.08 | **6.44E-06** | -0.04 | 2.04E-02 |
| W -> S | *P. sennae* | -0.09 | **4.02E-06** | -0.07 | **1.49E-04** | 0.03 | 6.50E-02 |

**Supplementary Table 14.** Summary of the multiple linear model analysis with log(ω) as the dependent variable and with GC-content at third position (GC3), observed effective number of codons (ENC_obs_) and normalised difference between observed and expected effective number of codons (ENC_diff_) as explanatory variables. The summary estimates and confidence intervals for each branch and variable are included, as well as adjusted R^2^, residual standard error (Res. Std. Error) and F-statistic for each model.

| **Log(ω)** | ***B. mori*** | ***P. machaon*** | ***L. accius*** | ***L. sinapis*** | ***P. sennae*** | ***C. cecrops*** | ***D. plexippus*** | ***H. melpomene*** |
| --- | --- | --- | --- | --- | --- | --- | --- | --- |
| **Estimate (CI)** |  |  |  |  |  |  |  |  |
| **(Intercept)** | -2.611 *** | -2.672 *** | -2.745 *** | -2.665 *** | -2.984 *** | -2.584 *** | -2.843 *** | -2.946 *** |
|  | (-2.645,-2.577) | (-2.708,-2.636) | (-2.782,-2.707) | (-2.700,-2.630) | (-3.026,-2.942) | (-2.619,-2.548) | (-2.883,-2.804) | (-2.986,-2.906) |
| **GC3** | -0.073 *** | -0.088 *** | -0.143 *** | -0.072 *** | -0.126 *** | 0.029 | -0.070 *** | -0.174 *** |
|  | (-0.108,-0.038) | (-0.126,-0.051) | (-0.181,-0.105) | (-0.113,-0.030) | (-0.170,-0.082) | (-0.008,0.066) | (-0.111,-0.028) | (-0.234,-0.114) |
| **ENC_obs_** | -0.062 ** | -0.120 *** | -0.091 *** | -0.084 *** | -0.088 *** | -0.086 *** | -0.095 *** | -0.076 * |
|  | (-0.103,-0.022) | (-0.162,-0.079) | (-0.134,-0.048) | (-0.132,-0.036) | (-0.139,-0.038) | (-0.124,-0.047) | (-0.143,-0.047) | (-0.141,-0.012) |
| **ENC_diff_** | -0.046 * | -0.101 *** | -0.103 *** | -0.118 *** | -0.090 *** | -0.086 *** | -0.069 ** | -0.094 *** |
|  | (-0.087,-0.006) | (-0.142,-0.059) | (-0.146,-0.060) | (-0.162,-0.074) | (-0.141,-0.039) | (-0.123,-0.048) | (-0.117,-0.021) | (-0.147,-0.041) |
| **R^2^/R^2^adjusted** | 0.012 / 0.011 | 0.024 / 0.023 | 0.026 / 0.025 | 0.023 / 0.022 | 0.021 / 0.020 | 0.012 / 0.011 | 0.013 / 0.012 | 0.047 / 0.046 |
| **Res. Std. Error** | 0.925 | 0.984 | 1.029 | 0.956 | 1.151 | 0.968 | 1.075 | 1.094 |
| **F Statistic** | 11.600*** | 24.003*** | 25.969*** | 22.155*** | 20.606*** | 11.750*** | 12.459*** | 47.463*** |
| **Observations 2881 (df = 3; 2877) ** p<0.05   ** p<0.01   *** p<0.001*** | | | | | | | | |

# Supplementary analysis

Candidate genes under positive selection in *L. sinapis*

We identified a set of candidate genes under positive selection in the *Leptidea* lineage. The aims of this analysis were i) to compare the base composition between conserved, global and positively selected gene sets and ii) to characterize gene classes under positive selection that could be associated with the unique properties of this particular lineage, i.e. a spectacular intraspecific variation in chromosome numbers and segregation of chromosome fission/fusion polymorphisms in some populations (Dincă et al. 2011; Lukhtanov et al. 2011, 2018).

Methods

For information on genome assemblies, multiple sequence alignment and filtering, see main text. To characterize a set of potentially positively selected genes in wood whites, the branch-site model in codeml in PAML v. 4.9e was applied (Zhang *et al.*, 2005), using *L. sinapis* as the foreground branch. The model allows ω to vary among branches and sites in four different site classes; i) ω < 1 in all branches, ii) ω = 1 in all branches, iii) ω < 1 in background branches and > 1 in the foreground branch, and finally, iv) ω = 1 in background branches > 1 in the foreground branch. The null model reflects the neutral expectations and allows only three site classes; i) ω < 1 in all branches, ii) ω = 1 in all branches, and, iii) ω < 1 in the background branches and = 1 in the foreground branch (Yang 2007). The two models were compared for each respective gene by applying likelihood ratio tests, including false discovery rate (FDR) correction (adjusted p-value = 0.05) for multiple testing (Thissen et al. 2002).

Gene ontology (GO) enrichment analyses were performed to detect potential overrepresentation of specific functional categories in the classes biological process, molecular function and cellular component were quantified using PantherGO (Mi et al. 2019). The Fisher’s exact test implemented in the software evaluates over- or underrepresentation of ontology terms associated with the candidate genes as compared to the aligned gene set included in the analysis. The test statistic for each gene set was corrected for multiple testing with FDR correction (adjusted p-value < 0.05), as implemented in the software.

*Results*

We found 41 genes with specific codons showing evidence for positive selection in the *Leptidea* lineage. The average GC content in candidate genes under positive selection was significantly higher (50.9 +/- 5.1%) than in the aligned gene set (45.7 +/- 6.5%; *W* = 123,531, p-value = 2.2*10^-7^). The pattern was consistent across all three codon positions, but the difference was largest in the third codon position (Supplementary Analysis Figure 1a). The higher GC-content was a consequence of an increase in G- and C-ending codons, and not significant differential utilization of specific codon triplets (Supplementary Analysis Figure 1b). The candidate genes under selection in *Leptidea* were also significantly longer than the genes in the aligned gene set (mean 4,283 +/- 4,100 bp; *W* = 140,146, p-value = 1.84*10^-13^). Higher GC-content in genes under rapid sequence evolution have been observed in mammals and reptiles (Huttener et al. 2019). The length of exons and GC-content is positively correlated in vertebrates and bacteria, so the increased GC-content could be a consequence of the sequence length (Oliver & Marín 1996). An alternative, but not exclusive explanation, is that the strength of GC-biased gene conversion (gBGC) varies across genes in *Leptidea*, potentially as an effect of variation in double-strand break frequencies between genomic regions (Marais 2003). If so, it is possible that gBGC will result in substitution patterns that mimic positive selection in some particular genes, actually leading to erroneous inference of positive selection (Berglund et al. 2009). We cannot rule out that technical issues like sequencing biases (Benjamini & Speed 2012) or undetected multiple hits (Venkat et al. 2018) to some extent affect the results of base composition and evolutionary rate analysis.

There was no significant overrepresentation of specific functional categories among the positively selected genes in *Leptidea*. However, there were several candidate genes within this set with functions of interest for understanding lineage specific characteristics in *Leptidea* (Supplementary Analysis Table 1). Two of the candidate genes were associated with chromosome stability and segregation and are therefore of interest for further analysis aiming at understanding meiotic events and the mechanistic underpinnings of karyotype instability (Lukhtanov et al. 2018, 2020). Java no jive or *jnj*/CG5524 is homologous to SMC6 in humans, one of six protein complexes involved in structural maintenance of chromosomes in eukaryotes (Hirano 2002). The function of SMC6 is not fully known but it promotes double-strand break repair associated to homologous recombination and could possibly be involved in telomeric elongation by recombination in humans (Potts & Yu 2007; Potts et al. 2006) and *east* is important for correct chromosome segregation during meiosis (Wasser & Chia 2003).

**a** **b**


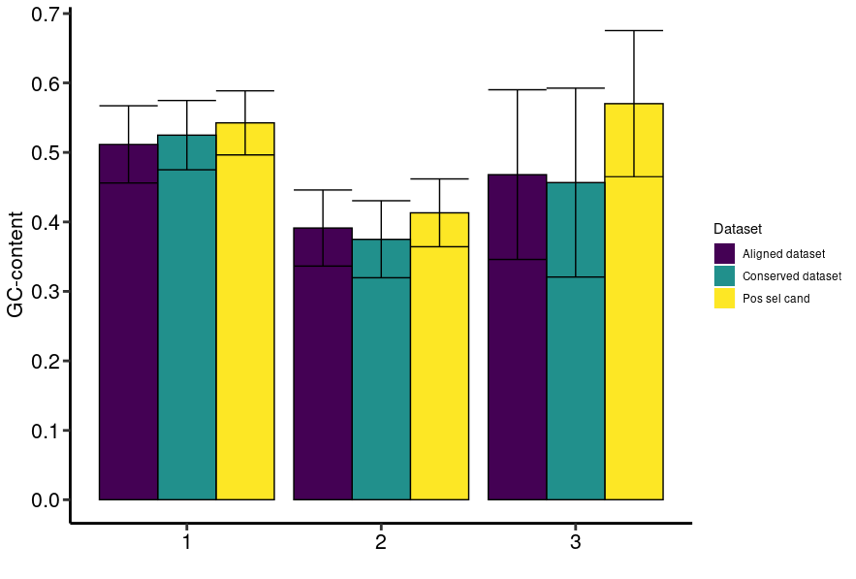

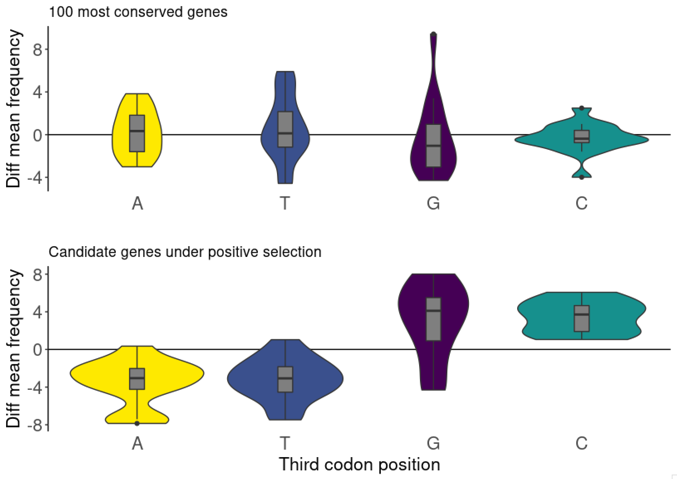


**Supplementary Analysis Figure 1.**

a) Barplot of GC-content in the different codon positions in the aligned geneset (dark blue), the 100 most conserved genes (green) and the candidate genes under positive selection in the Leptidea lineage (yellow). b) Difference in codon usage frequency between the aligned geneset and 100 most conserved genes (top), and between the aligned geneset and candidate genes under positive selection in the Leptidea lineage (bottom), grouped by nucleotide in third codon position.

**Supplementary Analysis Table 1.**

List of gene functions associated to candidate genes under selection in the *Leptidea* lineage. The genes are sorted after *Leptidea sinapis* gene-ID. Gene name is the gene symbol for the homologue in *D. melanogaster,* as is the geneID from FlyBase or Uniprot. The origin of the description and/or GO-term is noted (Source).

| **GeneID *L. sinapis*** | **Gene name** | **Flybase/ UniProt** | **Source** | **Description** | **GO-term** |
| --- | --- | --- | --- | --- | --- |
| leptidea_sinapisT00000000585 | jnj | FBgn0266282 | PantherGO | Java no jive, Smc6, structural maintenance of chromosomes protein6 | DNA metabolism protein |
| leptidea_sinapisT00000001028 |  | FBgn0037944 | NCBI | Unknown, ortholog to human ring finger, predicted to enable zinc ion binding activity |  |
| leptidea_sinapisT00000001034 | CG13397 | FBgn0014417 | PantherGO | Unknown, interacts with dsx, possibly enzyme involved in (HS-) GAG-metabolism |  |
| leptidea_sinapisT00000001077 | Dlg5 | FBgn0032363 | PantherGO | Discs large 5, isoform A | Anatomical structure morphogenesis(GO:0009653) |
| leptidea_sinapisT00000001247 | Ac78C | FBgn0024150 | PantherGO | Adenylyl cyclase 78C, isoform E | Purine ribonucleotide biosynthetic process(GO:0009152); regulation of cAMP-mediated signaling (GO:0043949) integral component of plasma membrane (GO:0005887); |
| leptidea_sinapisT00000001399 |  |  | BLAST | Calpains are ubiquitous, well-conserved family of calcium-dependent cysteine proteases |  |
| leptidea_sinapisT00000001613 | Rel | FBgn0014018 | PantherGO | Nuclear factor NF-kappa-B p110 subunit;Rel; P53-like transcription factor(PC00253) | RNA Polymerase II transcription factor activity, sequence-specific DNA binding(GO:0000981); chromatin binding(GO:0003682) |
| leptidea_sinapisT00000003245 | CG5167 | FBgn0038038 | PantherGO | CG5167;DmelCG5167; Saccharopine dehydrogenase-like oxidoreductase | Glycolipid biosynthetic process (GO:0009247) plasma membrane (GO:0005886) |
| leptidea_sinapisT00000005079 | Baldspot | FBgn0260960 | PantherGO | Elongation of very long chain fatty acids protein; acyltransferase(PC00042) | Very long-chain fatty acid metabolic process (GO:0000038) integral component of endoplasmic reticulum membrane (GO:0030176); |
| leptidea_sinapisT00000005195 | P58IPK | FBgn0037718 | PantherGO/ FlyBase | LD25575p; Chaperone binding |  |
| leptidea_sinapisT00000007350 | Oatp26F | FBgn0051634 | PantherGO | Solute carrier organic anion transporter family member | Organic anion transporter |
| leptidea_sinapisT00000007562 | HPS4 | FBgn0034261 | PantherGO | Hermansky-Pudlak syndrome 4 , isoform A; autosomal recessive genetic disease, decreased pigmentation (albinism) | Biogenesis of lysosomal organelles complex 3 subunit 2 |
| leptidea_sinapisT00000007662 | HnRNP |  | NCBI | Unknown Heterogeneous nuclear ribonucleoprotein |  |
| leptidea_sinapisT00000007870 | CG14967/hobbit | FBgn0035420 | FlyBase | Involved in positive regulation of intracellular transport. |  |
| leptidea_sinapisT00000007988 | CG9004 | FBgn0035336 | PantherGO | Nucleolar MIF4G domain-containing protein 1 homolog | mRNA processing factor (PC00147); nuclease(PC00170) RNA binding (GO:0003723) ribosomal small subunit biogenesis (GO:0042274) nucleolus (GO:0005730) |
| leptidea_sinapisT00000008411 |  |  | BLAST | Phosphoinositide 3-kinase regulatory subunit 4 isoform X1 |  |
| leptidea_sinapisT00000009333 | ItgaPS4 | FBgn0034005 | PantherGO | Integrin alpha-PS4 | Cell adhesion(GO:0007155) Integrin signalling pathway->Integrin alpha |
| leptidea_sinapisT00000010098 | RpI135 | FBgn0003278 | PantherGO | DNA-directed RNA polymerase I subunit RPA2; DNA-directed RNA polymerase(PC00019);nucleotidyltransferase(PC00174) | RNA polymerase I activity (GO:0001054) DNA-directed RNA polymerase I complex (GO:0005736); nuclear chromatin (GO:0000790) General transcription by RNA polymerase I |
| leptidea_sinapisT00000010336 |  |  | BLAST | endothelin-converting enzyme homolog |  |
| leptidea_sinapisT00000010522 | Gart | FBgn0000053 | PantherGO | Trifunctional purine biosynthetic protein adenosine-3; hydrolase(PC00121);ligase(PC00142);transferase(PC00220) | Ligase activity(GO:0016874) purine nucleobase biosyntheticprocess (GO:0009113); purine nucleotide biosynthetic process (GO:0006164) cytosol (GO:0005829) |
| leptidea_sinapisT00000010634 | EloA | FBgn0039066 | PantherGO | Transcription elongation factor B polypeptide 3 | Transcription elongation complex |
| leptidea_sinapisT00000011763 | CG15439 | FBgn0031606 | FlyBase | unknown PHD (plant homeodomain) zink-finger containing protein, Predicted to enable histone binding activity |  |
| leptidea_sinapisT00000013806 | apolpp | FBgn0087002 | PantherGO | Apolipophorins | Lipid transporter activity (GO:0005319) |
| leptidea_sinapisT00000014483 | cic | Q9U1H0 | BLAST | PREDICTED: Bombyx mori putative transcription factor capicua |  |
| leptidea_sinapisT00000014587 | fred | FBgn0051774 | PantherGO | Friend of echinoid, isoform H; Nephrin-related |  |
| leptidea_sinapisT00000014846 | stan | FBgn0024836 | PantherGO | Protocadherin-like wing polarity protein stan | Cadherin; Cell adhesion(GO:0007155) |
| leptidea_sinapisT00000014968 | CPR127 |  | BLAST | CPR127 cuticular protein RR-2 motif 127, B. mori |  |
| leptidea_sinapisT00000015014 | RhoGAP93B | FBgn0038853 | PantherGO | FI04035p; FI04035P (PTHR45876:SF8) | GTPase activator activity |
| leptidea_sinapisT00000015090 | TNK1 |  | BLAST/ UniProt | non-receptor (non-transmembrane) tyrosine-protein kinase TNK1 Involved in negative regulation of cell growth. |  |
| leptidea_sinapisT00000015480 | lachesin |  | BLAST | Lachesin (Lac), a cell surface protein, immunoglobulin superfamily protein |  |
| leptidea_sinapisT00000015546 | mt:CoI | FBgn0013674 | PantherGO | Cytochrome c oxidase subunit 1 | Oxidase (PC00175) |
| leptidea_sinapisT00000015572 | CG4658 | FBgn0032170 | NCBI/ Google | Unknown, possibly involved in temperature regulation |  |
| leptidea_sinapisT00000015830 | FASN1 | FBgn0283427 | PantherGO | Fatty acid synthase 1, isoform C;FASN1; | Hydrolase activity, acting on ester bonds(GO:0016788);ligase activity(GO:0016874);methyltransferase activity(GO:0008168);transferase activity, transferring acyl groups(GO:0016746) fatty acid biosynthetic process(GO:0006633) |
| leptidea_sinapisT00000015907 | CG43209 | FBgn0262845 | PantherGO | Uncharacterized protein;DmelCG43209; |  |
| leptidea_sinapisT00000016198 | east | FBgn0261954 | PantherGO/ FlyBase | EG:133E12.4 protein; enhanced adult sensory threshold (east) involved in chemosensory behavior, chromosome segregation, cell survival and muscle development. |  |
| leptidea_sinapisT00000016847 | CG5033 | FBgn0028744 | PantherGO | Ribosome biogenesis protein BOP1 homolog | Ribonucleoprotein complex binding(GO:0043021) maturation of LSU-rRNA from tricistronic rRNA transcript (GO:0000463) 90S preribosome(GO:0030686); preribosome, large subunit precursor(GO:0030687);t-UTP complex(GO:0034455) |
| leptidea_sinapisT00000016902 |  |  | BLAST | PREDICTED: Bombyx mori sorting nexin-14-like (LOC105842520), mRNA |  |
| leptidea_sinapisT00000017159 | stc | FBgn0001978 | PantherGO | Protein shuttle craft;stc; Transcriptional repressor NF-X1, zinc finger transcription factor(PC00244), | RNA polymerase II regulatory region sequence-specific DNA binding(GO:0000977) negative regulation of transcription by RNA polymerase II(GO:0000122);transcription by RNA polymerase II(GO:0006366) |
| leptidea_sinapisT00000018045 | Cad88C | FBgn0038247 | PantherGO | Cadherin 88C | Cell adhesion(GO:0007155) integral component of plasma membrane(GO:0005887) Wnt signaling pathway |
| leptidea_sinapisT00000019133 | nocte | FBgn0261710 | PantherGO/ EMBL/EBI | LP18708p;esterase(PC00097); transcription factor(PC00218) Temperature compensation of the circadian clock, entrainment of circadian clock | Histidine kinase receptor of two-component system |
| leptidea_sinapisT00000020691 | CG5728 | FBgn0039182 | PantherGO | CG5728;DmelCG5728; Protein RRP5 homolog, nucleic acid binding(PC00171) | RNA binding(GO:0003723) small-subunit processome(GO:0032040);t-UTP complex(GO:0034455) |

References

Benjamini Y, Speed TP. 2012. Summarizing and correcting the GC content bias in high-throughput sequencing. Nucleic Acids Research. 40:e72–e72. doi: 10.1093/nar/gks001.

Berglund J, Pollard KS, Webster MT. 2009. Hotspots of Biased Nucleotide Substitutions in Human Genes. PLOS Biology. 7:e1000026. doi: 10.1371/journal.pbio.1000026.

Dincă V, Lukhtanov VA, Talavera G, Vila R. 2011. Unexpected layers of cryptic diversity in wood white *Leptidea* butterflies. Nat Commun. 2:324. doi: 10.1038/ncomms1329.

Hirano T. 2002. The ABCs of SMC proteins: two-armed ATPases for chromosome condensation, cohesion, and repair. Genes Dev. 16:399–414. doi: 10.1101/gad.955102.

Huttener R et al. 2019. GC content of vertebrate exome landscapes reveal areas of accelerated protein evolution. BMC Evolutionary Biology. 19:144. doi: 10.1186/s12862-019-1469-1.

Lukhtanov VA et al. 2018. Versatility of multivalent orientation, inverted meiosis, and rescued fitness in holocentric chromosomal hybrids. Proc Natl Acad Sci U S A. 115:E9610–E9619. doi: 10.1073/pnas.1802610115.

Lukhtanov VA, Dincă V, Friberg M, Vila R, Wiklund C. 2020. Incomplete Sterility of Chromosomal Hybrids: Implications for Karyotype Evolution and Homoploid Hybrid Speciation. Front. Genet. 11:583827. doi: 10.3389/fgene.2020.583827.

Lukhtanov VA, Dincă V, Talavera G, Vila R. 2011. Unprecedented within-species chromosome number cline in the Wood White butterfly *Leptidea sinapis* and its significance for karyotype evolution and speciation. BMC Evol Biol. 11:109. doi: 10.1186/1471-2148-11-109.

Marais G. 2003. Biased gene conversion: implications for genome and sex evolution. Trends Genet. 19:330–338. doi: 10.1016/S0168-9525(03)00116-1.

Mi H, Muruganujan A, Ebert D, Huang X, Thomas PD. 2019. PANTHER version 14: more genomes, a new PANTHER GO-slim and improvements in enrichment analysis tools. Nucleic Acids Research. 47:D419–D426. doi: 10.1093/nar/gky1038.

Oliver JL, Marín A. 1996. A relationship between GC content and coding-sequence length. J Mol Evol. 43:216–223. doi: 10.1007/BF02338829.

Potts PR, Porteus MH, Yu H. 2006. Human SMC5/6 complex promotes sister chromatid homologous recombination by recruiting the SMC1/3 cohesin complex to double-strand breaks. EMBO J. 25:3377–3388. doi: 10.1038/sj.emboj.7601218.

Potts PR, Yu H. 2007. The SMC5/6 complex maintains telomere length in ALT cancer cells through SUMOylation of telomere-binding proteins. Nat Struct Mol Biol. 14:581–590. doi: 10.1038/nsmb1259.

Thissen D, Steinberg L, Kuang D. 2002. Quick and Easy Implementation of the Benjamini-Hochberg Procedure for Controlling the False Positive Rate in Multiple Comparisons. Journal of Educational and Behavioral Statistics. 27:77–83. doi: 10.3102/10769986027001077.

Venkat A, Hahn MW, Thornton JW. 2018. Multinucleotide mutations cause false inferences of lineage-specific positive selection. Nat Ecol Evol. 2:1280–1288. doi: 10.1038/s41559-018-0584-5.

Wasser M, Chia W. 2003. The Drosophila EAST protein associates with a nuclear remnant during mitosis and constrains chromosome mobility. J Cell Sci. 116:1733–1743. doi: 10.1242/jcs.00379.

Yang Z. 2007. PAML 4: phylogenetic analysis by maximum likelihood. Mol Biol Evol. 24:1586–1591. doi: 10.1093/molbev/msm088.
